# Safe traveling in public transport amid COVID-19

**DOI:** 10.1126/sciadv.abg3691

**Published:** 2021-10-22

**Authors:** Donggyun Ku, Chihyung Yeon, Seungjae Lee, Kyuhong Lee, Kiyeon Hwang, Yuen Chong Li, Sze Chun Wong

**Affiliations:** 1 Department of Transportation Engineering, University of Seoul, Seoul, South Korea.; 2 Inhalation Toxicology Center for Airborne Risk Factors, Korea Institute of Toxicology, Daejeon, South Korea.; 3 Department of Urban Engineering, Hongik University, Seoul, South Korea.; 4 Department of Civil Engineering, The University of Hong Kong, Hong Kong.; 5 Guangdong–Hong Kong–Macau Joint Laboratory for Smart Cities, Hong Kong, China.

## Abstract

Several intensive policies, such as mandatorily wearing masks and practicing social distancing, have been implemented in South Korea to prevent the spread of the novel coronavirus disease (COVID-19). We analyzed and measured the impact of the aforementioned policies by calculating the degree of infection exposure in public transport. Specifically, we simulated how passengers encounter and infect each other during their journeys in public transport by tracking movements of passengers. The probabilities of exposure to infections in public transport via the aforementioned preventive measures were compared by using the Susceptible, Exposed, Infected, and Recovered model, a respiratory infectious disease diffusion model. We determined that the mandatory wearing of masks exhibits effects similar to maintaining 2-m social distancing in preventing COVID-19. Mandatory wearing of masks and practicing social distancing with masks during peak hours reduced infection rates by 93.5 and 98.1%, respectively.

## Teaser

Safe travel in public transport amid COVID requires wearing a mask properly, showing effects similar to 2-m social distancing.

## INTRODUCTION

The world is currently facing the enormous challenge of overcoming the outbreak of coronavirus disease (COVID-19). In most countries, where COVID-19 cases are growing at alarming rates, local governments have tried to implement lockdowns and social distancing schemes to slow the spread of COVID-19. Furthermore, evidence has shown that staying at home, reducing nonessential travel, and maintaining a safe distance from others are of utmost importance to prevent the spread of the disease (*1*). Although South Korea is one of the first countries to be affected by the COVID-19 outbreak, the containment measures adopted by the government were successful in controlling the disease via a combination of widespread testing, aggressive contact tracing, travel restrictions, quarantine, and social distancing measures. However, individuals are increasingly becoming tired of isolation and quarantine as these measures have been extended. Thus, many individuals are becoming impatient, thereby relaxing social distancing and resuming their daily life activities.

In South Korea, the major public transportation systems consist of urban rails and buses, which provide convenient and safe travel services for all the citizens. Seoul, the capital of South Korea, has been one of the world’s fastest-growing cities. In Seoul, rail and bus services transport approximately 65% of all passengers. Hence, Seoul has one of the highest market shares of public transportation in the world (*2*). Since the COVID-19 outbreak, the demand to travel on public transportation service has dropped sharply, especially after the government raised the infectious disease alert level to “serious” on February 23 (*3*). To safely reopen the economy, access to public transportation service is critical and vulnerable. Hence, guidelines have been issued by different governments, including that of South Korea, to aid people in traveling safely during the COVID-19 pandemic. The guidelines include the following measures: maintain safe distance from others to the maximum possible extent, wear a face covering, travel at off-peak times, and avoid crowds in public places. However, traveling using public transportation is considered risky because COVID-19 is a highly contagious disease and infected people may not exhibit any symptoms (*4*). Thus, the reasons for the existing anxiety is justified by the vulnerability of public transportation to COVID-19 infection (*5*). Sun *et al.* (*6*) proved that public transit can be a source of transmission of the infectious epidemic. Furthermore, Mo *et al.* (*7*) concluded that public transit is a critical factor of infection. In addition, several studies concluded that the improvement in urban resilience, such as reconsideration of space allocation methods in transportation systems (*8*), is required.

## RESULTS AND DISCUSSION

**Fig. 1.**
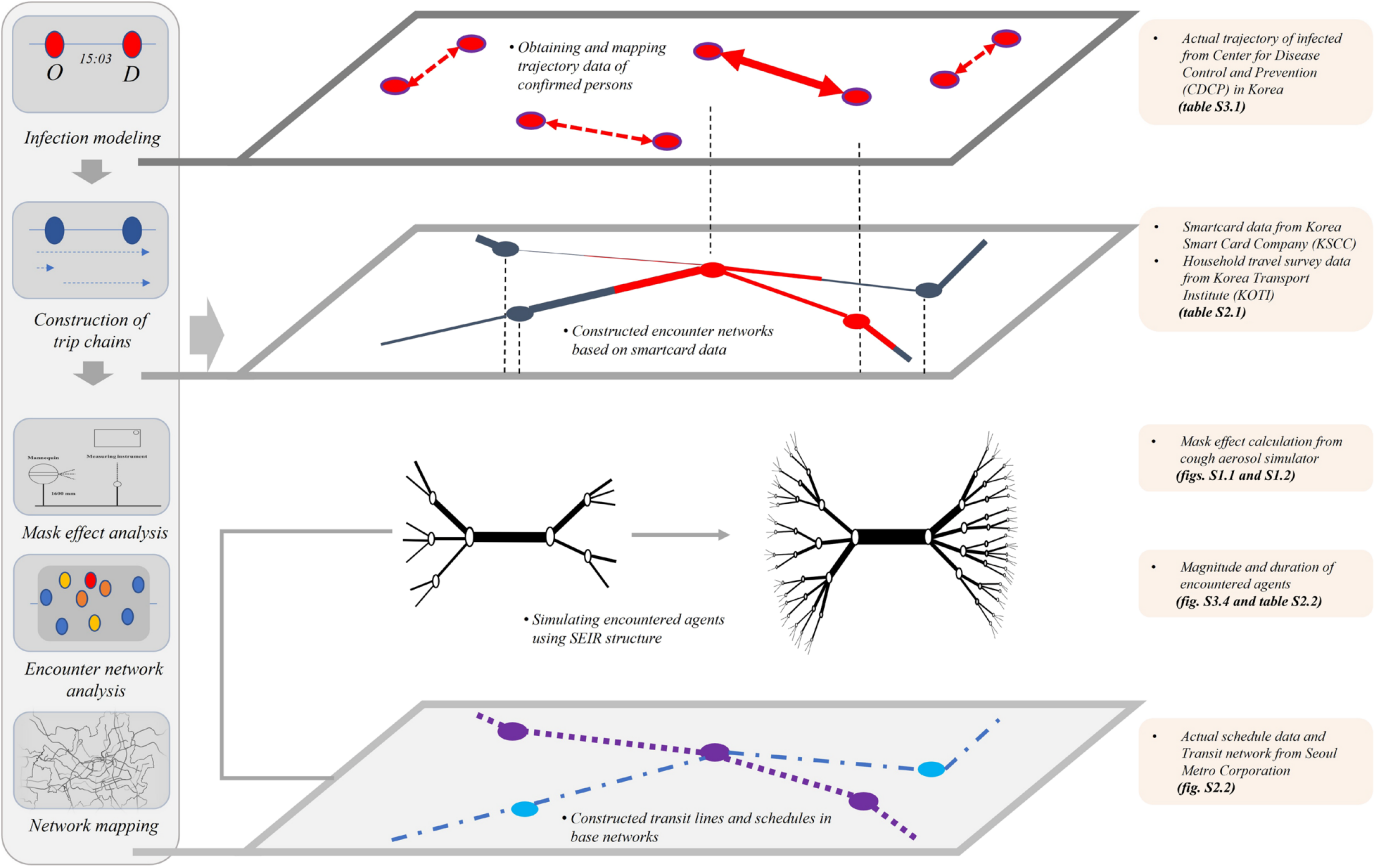
Conceptual diagram of the encounter network based on actual data. This figure shows the conceptual diagram of the encounter network based on real infected people. First, agent-based modeling was used to assign a chain of trips by individual agents according to their smart card data. The resulting model allowed us to track the movements of these individual agents. We then used the tracking data to compute the degree and duration of contact experienced by each agent. Through these steps, the level of congestion in public transportation was calculated, as shown in the diagram below.

Generally, COVID-19 is considered to spread mainly through close contact between individuals. Hence, certain individuals without any symptoms can spread the virus. Thus, it is critical to understand the travel patterns and activities of infected individuals in protecting and controlling the spread of COVID-19. In transportation research, various studies used different networks or frameworks, such as Neural Network (*9*), Euclidean Network (*10*), and Reverse Logistics Network (*11*), to simulate the trip characteristics and travel patterns of passengers in public transportation. Alternatively, Sun *et al.* (*6*) studied the public transportation passenger encounter pattern via an Encounter Network by using smart card data (*12*). We extended the work by Sun *et al.* (*6*) by proposing a time-varying weighted Public Transportation Encounter Network that modeled the COVID-19 infectious process in public transportation. First, we used the smart card data to implement the traveler’s trip chain and performed traffic assignment under the agent-based model (*13*). In this study, the records of two databases, including the smart card data and real data on infected individuals, were adopted for constructing and analyzing the model. Information on smart card data includes the identifier ID of the transit user, location, boarding time, and disembarking time. Based on the limitation of the technique, smart card data were composed of only original-destination (OD) pairs. Thus, the movement path information of individual users was not provided. Therefore, in this study, we performed a transit assignment process to estimate the shortest movement path of each individual in public transportation with respect to time zone, congestion by section (see Fig. 1 and Supplementary Materials, section 2), and the encounter network (*13*). Subsequently, contact with other public transportation users was analyzed when emulating the actual infected people’s movements on the public transportation network in Seoul. Based on the framework of multi-agent transport simulation (MATSim) (*14*), the urban transportation network in metropolitan areas plays an essential role in commutes and movements between frequently visited locations. The trips’ characteristics showed a repeated mobility pattern of regularity and thereby following simple reproducible patterns (*15*). Thus, the familiar stranger group, which a specific public transportation user frequently encounters, was analyzed. Based on this framework, it was possible to simulate the spreading process of the COVID-19 virus in public transportation networks over a period of time. This simulation process showed how public transportation users are exposed to the virus during their trips (see Supplementary Materials, section 3).

In the current COVID-19 response, the countermeasure policies, such as wearing a mask and maintaining a social distance, are implemented. In South Korea, the use of masks is recommended to suppress the spread of COVID-19 virus among people. In public places, such as public transportation, either outside or inside, people are almost mandated to wear a mask. The emphasis on wearing a mask aids in reducing the spread of the virus (*8*). Existing studies proved that wearing N95 masks can block almost all mock viruses (*16*, *17*). Hence, widespread use of the mask can effectively suppress aerosol propagation. According to a previous study, the median protection factor of an unauthenticated mask worn by an untrained individual is in the range of 2.4 to 6.5, or its mask efficiency is the range of 58 to 85% (*16*, *17*). In addition, the initial penetration of quarantine masks (KF94), i.e., government-distributed quarantine masks, ranges from 0.622 to 1.698% and that of yellow sand mask (KF80) is 12.476% on average (*18*, *19*). This mask efficiency is the effect on inhalation when wearing the mask, and it is applied to the present analysis via a combination of the effects on the exhalation results. To determine the number concentrations and the reduction rate before and after wearing the mask, experiment and literature review were conducted in this study. On the basis of some previous studies (*20*, *21*, *22*, *23*), we prepared a 5% TiO_2_ Gamble solution to mimic pulmonary fluids and viral particles and converted it to cough aerosols using a mist generator. A mannequin with a human face was used to model mask-wearing, and a cough aerosol simulator (CAS) was used to measure the rate of reduction in cough aerosols brought about by mask-wearing. The mannequin’s face had a hole that sprayed aerosols, which was covered with a KF94 or KF80 mask, which are readily available in domestic pharmacies. The scope of this study was limited to examining the formation of cough aerosols and their blockage by a mask. Moreover, this study assumed that a mask is worn properly and fits perfectly (i.e., no leakage), and that there is no additional airflow. However, the effects of several other related factors must be considered, such as the type and design of mask, and a user’s head shape and wearing behavior, as these factors may cause aerosol leakage due if a mask is poorly fitted. In addition, the use of air conditioners and the effects of wind can alter the distribution of aerosols. The effects of these factors can all be examined in future studies. The limited number and type of experiments in this study means that there is uncertainty associated with our extrapolated findings. However, by performing experiments on exhalation effects and reviewing inhalation effects under the aforementioned assumptions, we have determined that mandatory mask-wearing results in COVID-19–preventive effects similar to those resulting from maintaining 2-m physical distancing (see Supplementary Materials, section 1).

Furthermore, the government has been responding to COVID-19 by implementing many measures, including the introduction of a work system adjusted to the social distancing policy. Because of these policies, people are under the belief that the spread of infectious diseases has been minimized. Apparently, wearing a mask and maintaining a social distance can substantially reduce infection in public transportation. Hence, investigation on testing the efficiency of wearing a mask and maintaining a social distance and that on the degree of protection provided by these measures in protecting against the virus in public transportation are essential. Thus, in this study, we conducted the path analysis of exposures to justify the effectiveness of wearing a mask and maintaining social distance in public transportation. In South Korea, the Centers for Disease Control and Prevention (CDCP) provides all activities and paths of infectious individuals via epidemiological investigations. We were able to emulate the movement paths of all infected individuals, especially their exact activities before they were diagnosed as infected until the time when they received confirmation or returned to hospital or were quarantined, by using smart card data and real data on infected individuals. In the study, we performed infection diffusion pathway analysis based on the individual encounter network analysis on the uses of public transportation by infected individuals in South Korea under two sets of simulations: the “uncontrolled” (without wearing a mask and maintaining no social distancing measures) and “controlled” conditions. The SEIR (Susceptible, Exposed, Infected, and Recovered) model was applied to perform the encounter network analysis (see Supplementary Materials, sections 3 and 4.2).

**Fig. 2.**
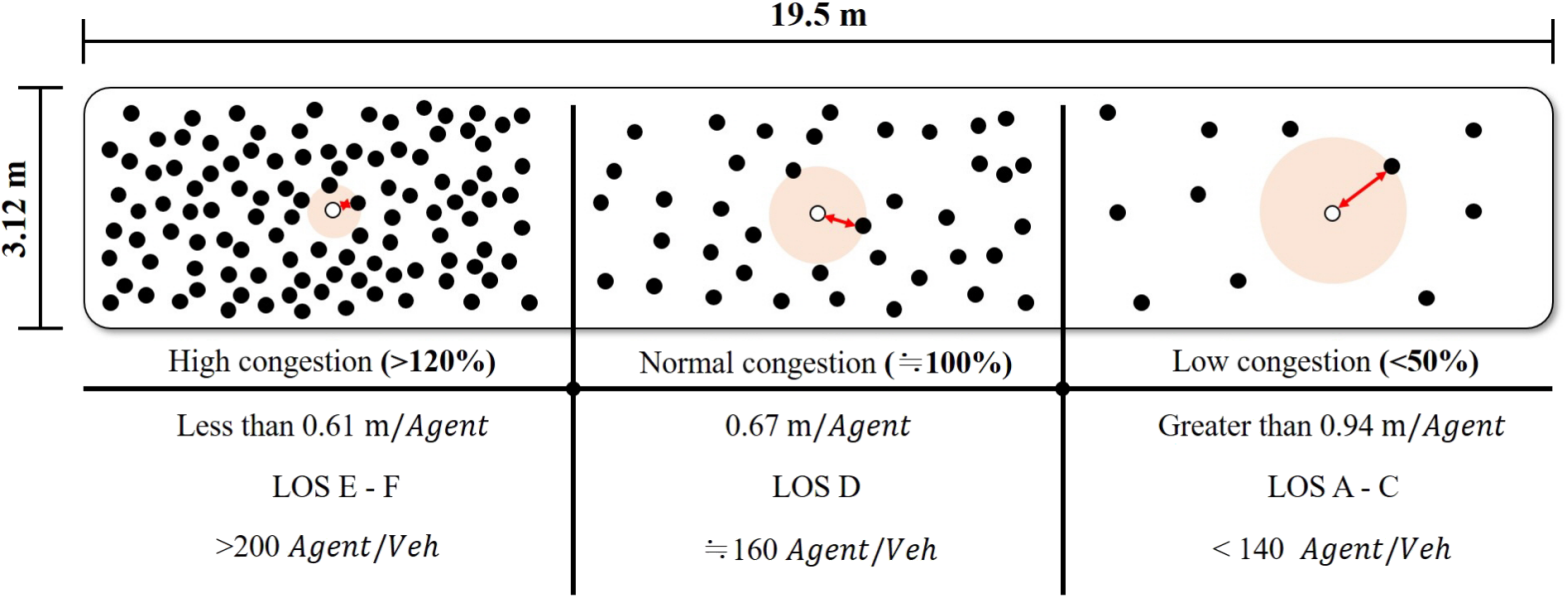
Determination of the average distance between individual agents by the level of congestion on public transport. This figure shows the average distance between individual agents in a metro compartment. Individual agents travel separately according to their travel time, origin, and destination. If these passengers board the same metro compartment, contact occurs. At this point, we calculated the congestion level according to the number of passengers in the metro compartment. Moreover, we calculated the average distance between individual agents according to the congestion level in the compartment by considering the area occupied by each agent. The result was then used to calculate the actual transmission probability for individual agents within a compartment. Congestion levels were defined as level of service (LOS). LOS E–F represents an average distance of no less than 0.61 m per agent, whereas LOS A–C represents an average distance greater than 0.94 m per agent. We tracked the number of people onboard public transport and calculated the average distance between individual agents and applied the result to the infection diffusion model.

**Fig. 3.**
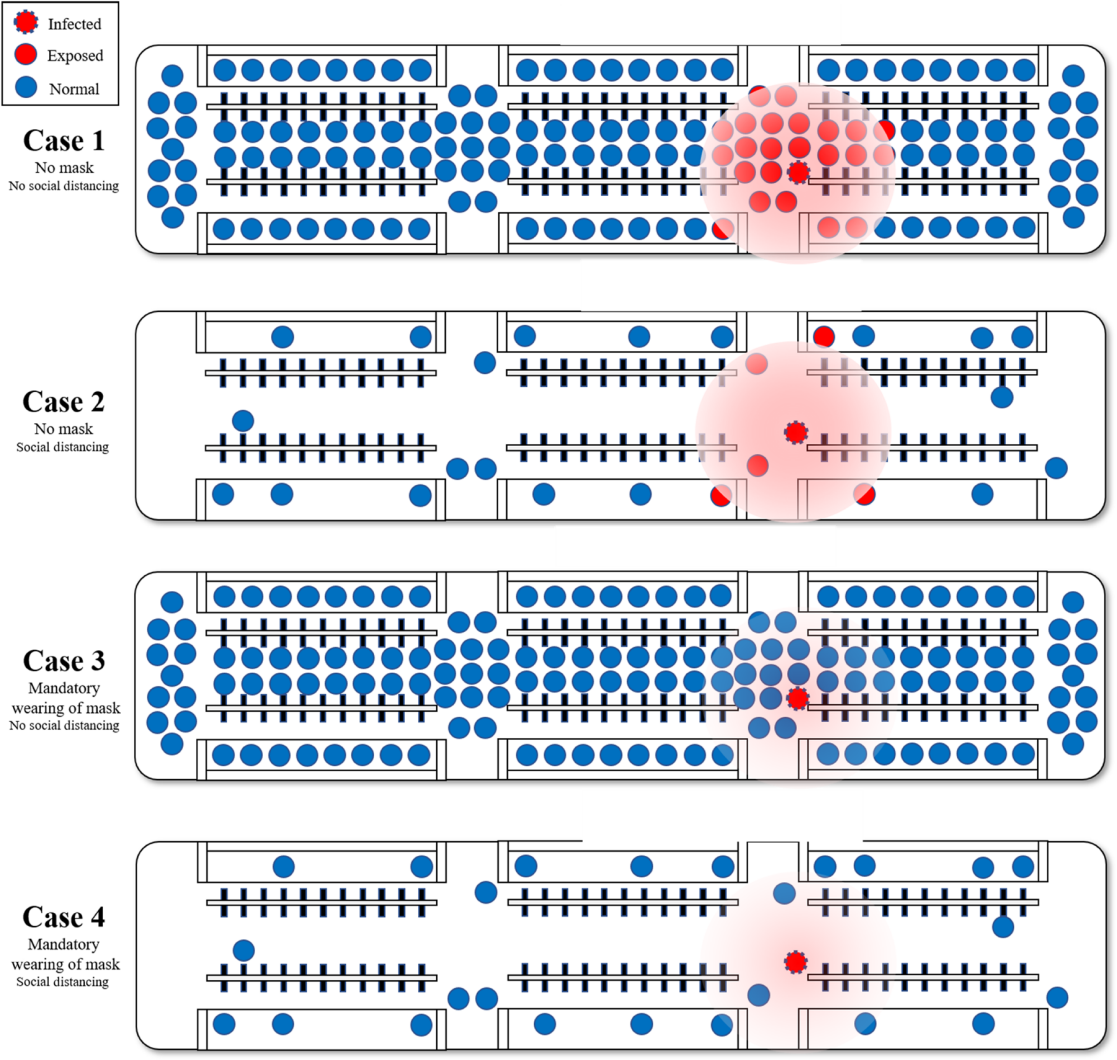
Prediction of the actual transmission probability according to quarantine policies such as mandatory mask wearing and social distancing. This figure shows the variation in the number of exposed agents based on the implementation of social distancing and mandatory mask wearing with respect to different case scenarios. Mask wearing reduces the distance the virus can spread, and social distancing increases the average distance between individual agents in a metro carriage, as in Fig. 1. These two policies contribute to reduced exposure. In case 1, agents are most likely to exist around the infected agent and are more likely to be exposed because of the high probability of the exposed agent because social distancing is not maintained and no mask is worn. In case 2, social distancing is maintained, and thus the probability of exposed agent is low. However, the probability of exposed agents around the infected agent is high, thereby resulting in some degree of exposed agent. In case 3, social distancing is not performed. However, the infected agent is not exposed to many agents because of the mask and thereby resulting in a sharp drop in the number of exposed agents. As shown in case 4, implementing both policies results in a sharp decrease in the exposed agent.

It is evident that large events and mass gatherings can contribute to the spread of COVID-19 via travelers who attend these events and carry the virus to new communities. Similarly, in public transportation, COVID-19 virus can spread as the number of encounters in the same space increases and as the duration of stay increases. As demonstrated by the public encounter network, variation in the demand for public transportation based on time zone can substantially affect the congestion levels. Hence, different congestion levels generated different encounter patterns in the public transportation network (see Supplementary Materials, section 2). Furthermore, in this study, it was demonstrated that the contact durations of infected individuals were closely related to the level of congestion in public transportation (see Figs. 2 and 3). The simulation reflected the real situation as to how the encounters between people decreased because of the containment measures implemented by the government with respect to controlling congestion levels.

**Fig. 4.**
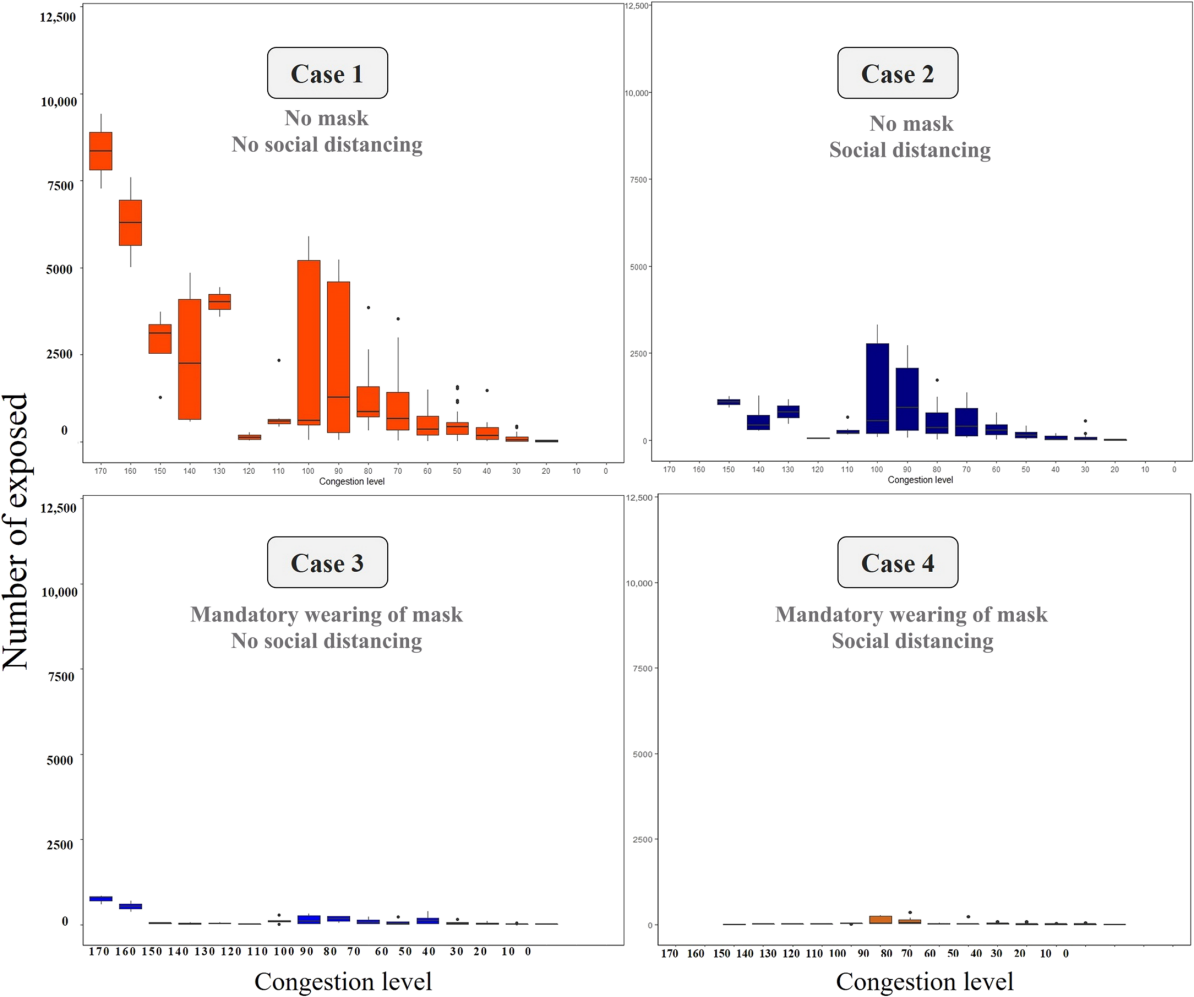
Trend of exposed agents based on the degree of congestion in each case. The graphs show the number of exposed agents based on different congestion levels for each case. First, the probability per person is not absolute and every individual agent’s probability of exposure varies depending on the actual probability of contact with the infected person, the congestion level in the public vehicle, and contact. The analysis calculated the probability of exposure for each individual agent over 1 day. The figure shows the average value generated by individual agents for each congestion level. The individual agent values cannot be presented because approximately 10 million journeys are made on the Seoul public transportation system per day. The average value is therefore calculated as the probability of exposure given the number of people exposed at different congestion levels. The standard deviations are also presented in the graph. In the case with social distancing (case 2), the upper limit of congestion is limited. Hence, the number of exposed agents decreases. When masks are worn (cases 3 and 4), the agent is less likely to be exposed (see Supplementary Materials, section 4.2).

**Fig. 5.**
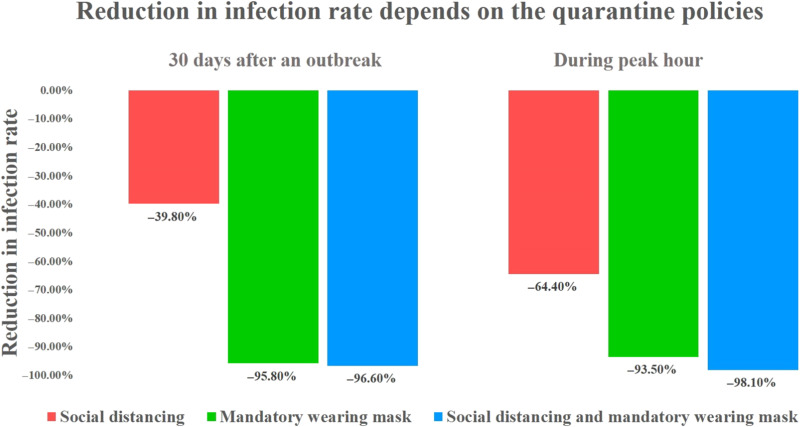
Reduction in infection rate by comparing a normal case with cases involving mandatory wearing of mask and social distancing with mask. The graph compares the reduction in infection rates based on the total number of agents exposed in the 30 days after an outbreak depends on the quarantine policies in force and during peak hours. In the cases involving mandatory mask wearing and social distancing with mask wearing, the infection rates are reduced by 95.8% (93.5% during peak hour) and 96.6% (98.1% during peak hour), respectively (see Supplementary Materials, section 4.2).

Furthermore, statistical verification was performed by grafting the case of exposure in public transportation to the theory of the SEIR model. In this simulation, we analyzed the degree of exposure to infecting COVID-19 virus by performing the analysis using the actual travel demand of the infected individuals. Public transportation passengers use smart card data, which provide information on departure locations, travel times, and arrival locations. These data and public transportation schedules were simulated to produce the traveling activities of passengers (see Supplementary Materials, section 2.2). The simulation revealed an interesting fact—passengers could repeatedly engage in a type of group traveling, called “familiar strangers,” by sharing common physical time and space in public transport facilities (see Supplementary Materials, section 2.3). Epidemic infection simulations, in conjunction with actual infection information provided by the Korea CDCP, were performed on groups of such familiar strangers to determine whether some passengers are exposed while traveling in public transport (see Supplementary Materials, section 3). This type of phenomenon can be determined via the network configuration wherein each agent affects other agents in the corresponding section. Each section has its own congestion level and proportionally affects the contact between each of the agents without their knowledge (see Supplementary Materials, section 2.3). Hence, it was proved that the probability of infection in public transportation is extremely low under the current public transportation scenario (see Supplementary Materials, section 4.2). Last, in the study, we conducted a verification of the difference in the degree of spread, congestion difference, encounter difference, and infection risk owing to the wearing of masks and the implementation of a social distancing policy. The verification results show a difference in the probability of exposure to infectious disease after 30-day outbreaks, depending on the quarantine policies. First, when social distancing is implemented without mandatory mask-wearing, such as in case 2, only the effect of reducing number and duration of encounters by reducing congestion can be reflected; this results in a 39.8% reduction. Second, when only wearing a mask is mandatory without social distancing, such as in case 3, a 95.8% reduction is observed because of the mask-wearing, even if congestions occur. Third, when both social distancing and mandatory mask-wearing are implemented, such as in case 4, the compounded benefits of distancing and mask-wearing result in a 96.6% reduction. In addition, the number of exposed individuals further decreased when social distancing is maintained in public transportation (see Fig. 5 and Supplementary Materials, section 4.2). However, the effect is not substantial (see Supplementary Materials, section 4.2). The effects of these two policies can be maximized during peak hours. Hence, the results indicated as to the level of congestion that should be maintained in public transportation to overcome the current COVID-19 epidemic. During peak hours, the number of exposed individuals decreased by 64.4%, when social distancing policies were implemented, 93.5%, when wearing of masks is mandatory, and 98.1%, when both polices are implemented (see Supplementary Materials, section 4.2, and Fig. 4). In conclusion, wearing a mask in public transportation can substantially reduce the number of exposures to the infection. This result is similar to that in a previous study that recommended a guideline of a 6-foot distance when wearing a mask to prevent indoor airborne transmission of COVID-19 (*24*). Social distancing is also shown to be effective. The model results reflect the current situation in South Korea, where most passengers wear masks consistently and properly (*25*) and there is a low probability of infection on public transport. Hence, the probability of exposure to infection decreased by wearing a mask and implementing social distancing policies, such as parallel work system and telecommuting.

## MATERIALS AND METHODS

### Analysis of inhalation and exhalation effects when wearing a mask

The effect of analysis on the mandatory wearing of a mask, one of the most important topics in this study, was conducted as follows. The mask effect analysis was performed by separating the effects on the inhalation and exhalation with the mask. The analysis was conducted based on the previously verified inhalation effects, and the results indicated that KF94 and KF80 masks exhibit 98.7 and 87.6% anti-inhalation effects (*18*, *19*), respectively.

To analyze exhalation effects with or without wearing a mask, we examined the change in particle concentration based on the distance from the source of aerosols due to coughing. To this end, we conducted an experiment to determine the degree of spread of imitated virus particles based on whether a mask was worn. To analyze the probability of infection according to the distance between individuals, the diffusion distance of aerosols based on the presence or absence of a mask was analyzed via direct experiments. The purpose of this study is to determine the degree of transport of infectious substances through aerosols based on the degree of response by individuals to the current policy of wearing masks in South Korea. In the experiment, we hypothesized the situation of spraying aerosols in air via cough. The sprayed aerosols can be divided into three categories, namely, droplets larger than 5 μm, micro-, and nano-sized aerosol droplets. It was observed that the droplets bulkier than 5 μm fall quickly on the ground and have an influence of approximately 2 m. However, the micro- and nano-sized aerosols remain in air for a longer time and can hence spread further. We examined the variation in concentrations of micro- and nano-sized particles with respect to the distance from the source of aerosol based on whether a mask was worn (see Supplementary Materials, section 1). We applied these experimental results to the diffusion model in case of public transportation.

### Encounter network analysis in public transportation

We implemented the trip chain based on smart card data and performed traffic assignment under the agent-based model. However, when using a smart card composed of only OD pairs, the movement path information of individual users is not provided. Hence, it is necessary to estimate the movement path of each individual with respect to time through the process of traffic assignment. On the basis of these traffic assignment steps, it is possible to implement an individual user’s movement route with respect to time zone, congestion of each section, and encounter network. We performed the traffic assignment in the shortest route in public transportation by referring to the manual that explained as to how to use the MATSim (*14*, *26*). The study was conducted with MATSim, which is a transportation simulation focused on transportation planning (*27*), and the user’s shortest route estimation (*28*). We set the congestion level for each section of the corresponding route with respect to time zone. Subsequently, based on the constructed result, we implemented the encounter network to calculate the contact duration. We analyzed this congestion with respect to each section and reflected it in the infection rate of each section. The area per person was assumed to be circle and converted into the distance between individuals. As a result, the 100% congestion-based agent is found to be about 1.3 m apart, and the 160% congestion-based distance is 1 m; except for the area occupied by the human body, there is virtually no empty space. Next, we combined the average distance between the individual agents and the changes in the particle distribution according to the distance. This combined value is reflected by the change (*C*) in the probability of an individual being infected. The ratio of the number of particles to the distance is expressed as a linear regression function. As the minimum distance between the individual agents is 0.51 m, we also set the minimum observational diffusion value to 0.5 m. Ratio *C* is set to 1 at a minimum distance of 0.5 m between agents; as the average distance increases, ratio *C* decreases according to the linear regression function (see Supplementary Materials, section 2).

### Path analysis of exposures

In South Korea, the CDCP provides the actual path of infection through epidemiological investigations. These data are provided in the form of OD pair for each time zone, similar to that in smart card data, from 00 days before the actual infected person receives confirmation of infection until the time when they receive confirmation or return to hospital or are in quarantine. In this study, we exploited data of real infected individuals available from the epidemiological survey data. We performed infection diffusion pathway analysis based on the analyzed individual encounter network. An SEIR structure was applied according to the contact duration calculated from the PEN in the public transportation network. The typical model of an epidemic can be divided into two categories: individual-based and degree-based approaches. The individual-based approach models epidemic transmission at the individual level, and the degree-based approach captures the infection process at the group level. Each group contains a set of nodes (individual) of the same level. In the PEN, we can characterize the behavior of human interaction at the individual level. Therefore, an individual-based framework was chosen in this study (see Supplementary Materials, section 3).
