# Supplementary material for: Safe traveling in public transport amid COVID-19
Source: Sci Adv. 2021 Oct 22;7(43):eabg3691. doi: 10.1126/sciadv.abg3691 (PMC8535823; doi:10.1126/sciadv.abg3691)
Supplement: Supplementary file 1 — Figs. S1 to S17 Tables S1 to S12 Legends for movies S1 to S3 [file sciadv.abg3691_sm.pdf]

Science Advances
AAAS

Supplementary Materials for

Safe traveling in public transport amid COVID-19

Donggyun Ku, Chihyung Yeon, Seungjae Lee*, Kyuhong Lee, Kiyeon Hwang,
Yuen Chong Li, Sze Chun Wong

*Corresponding author. Email: sjlee@uos.ac.kr

Published 22 October 2021, *Sci. Adv.* **7**, eabg3691 (2021)
DOI: 10.1126/sciadv.abg3691

The PDF file includes:

Figs. S1 to S17
Tables S1 to S12
Legends for movies S1 to S3

Other Supplementary Material for this manuscript includes the following:

Movies S1 to S3
Data file S1

1. Methods

1.1 Analysis of inhalation effects when wearing a mask.

The effect of analysis on the mandatory wearing of a mask, one of the most important topics in this study, was conducted as follows. The mask effect analysis was performed by separating the effects on the inhalation and exhalation with the mask. The analysis was conducted based on the previously verified inhalation effects, and the results indicated that KF94 and KF80 masks exhibit 98.7% and 87.6% anti-inhalation effects (*17*), respectively.

1.2 Analysis on the degree of diffusion of droplets with or without wearing a mask.

In this study, we examined the change in particle concentration based on the distance from the source of aerosols due to coughing. To this end, we conducted an experiment to determine the degree of spread of imitated virus particles based on whether a mask was worn. To analyze the probability of infection according to the distance between individuals, the diffusion distance of aerosols based on the presence or absence of a mask was analyzed via direct experiments. The purpose of this study is to determine the degree of transport of infectious substances through aerosols based on the degree of response by individuals to the current policy of wearing masks in South Korea.

In the experiment, we hypothesized the situation of spraying aerosols in air via cough. The sprayed aerosols can be divided into three categories, namely, droplets larger than 5 µm, micro-, and nano-sized aerosol droplets. It was observed that the droplets bulkier than 5 µm fall quickly on the ground and have an influence of approximately 2 m. However, the micro and nano sized aerosols remain in air for a longer time and can hence spread further. We examined the variation in concentrations of micro- and nano-sized particles with respect to the distance from the source of aerosol based on whether a mask was worn.

Fig. S1 illustrates a Cough Aerosol Simulator (CAS) for producing micro-sized cough aerosols. A spraying device (DIA-3530, Furupla, Japan) was used to produce aerosols. Additionally, an OPC (Optical Particle Counter, 11-D, Grimm, Germany)) was used as a measuring instrument to measure the aerosol size.

Some modifications were made to the Cough Aerosol Simulator (CAS) for generating nano-sized cough aerosols, as depicted in **Fig. S2** The CAS consisted of a mist generator (VMG, Vitals, Korea), compressed dry air supplier (LTC-7502, Ace power, China), buffer mixer, and two hydraulic valves (HPW2130, Autosigma, Korea) that selectively regulated two flow paths. It had also two ball flow controllers. One was for generation flow control (50 LPM, RMA-23-SSV, Dwyer, USA) and the other was for additional flow control to make total flow (300 LPM, LF401, Unicell, Korea). A total flow rate of 230 LPM was normally vented to the drain. Furthermore, 1.7 L were emitted in a cough duration of 0.44 s through the mouth of a mannequin. This condition was modeled using the sinusoidal function with an average cough flow (1.69 L) and maximum cough flow rate (6.01 L/sec) for influenza patients as observed in a previous study *(18).*

A mannequin was used to imitate a human face wearing a mask. The aerosol was sprayed through the mouth of the mannequin. KF94, KF80 masks were used considering that they are readily available in domestic pharmacies. The mass concentrations of micro-sized cough aerosol particles were measured according to the distance from mannequin before and after wearing the mask to estimate their Reduction rate.

Gamble solution representing interstitial deep fluid within the lungs was used to simulate the droplets produced by cough *(19)*. It was prepared by sequentially dissolving the following constituents in sterilized distilled water: 0.095 g/L magnesium chloride, 6.019 g/L sodium chloride, 0.298 g/L potassium chloride, 0.126 g/L disodium hydrogen phosphate, 0.063 g/L sodium sulfate, 0.368 g/L calcium chloride dihydrate, 0.574 g/L sodium acetate, 2.604 g/L sodium bicarbonate, and 0.097 g/L sodium citrate dihydrate. This reagent was supplied by Sigma-Aldrich (Mo, USA). The pH of solution was adjusted to pH 7.4 using 37% HCL (Acros Organics, NJ, USA) after the dissolution.

The SARS-CoV-2 has a diameter of about 70 ~ 90 nm. TiO2 particles (Merck, USA) were used to simulate virus particles because, in our previous study *(21),* the geometric mean diameter of ~ 80 nm was obtained from TiO2 distilled water solution spray. Accordingly, we prepared a 5% TiO2 Gamble solution to mimic the pulmonary fluid and virus particles and sprayed it using the mist generation illustrated in **Figs. S1 and S2.**

The experiments were performed in an exposure chamber with a length of 4 m, width of 3 m, and height of 2.4 m. The exposure chamber was maintained at a temperature of 22 °C and a relative humidity of 50%. During the exposure assessments, the number of ventilations was maintained at 0.5/hour to minimize airflow. The flow velocity was 20 cm/min. Between the exposure assessments, the number of ventilations was increased to 30/hour to clean the air inside the chamber and sufficiently reduce the background particle level of atmosphere in exposure changer. Clean air that had passed through the HEPA filter flowed from the top inlet to the bottom outlet.

1.3 Study limitations and suggestions for future research

The scope of this study was limited to examining the formation of cough aerosols and their blockage by a mask. Moreover, this study assumed that a mask is worn properly and fits perfectly (i.e., no leakage), and that there is no additional airflow. However, the effects of several other related factors must be considered, such as the type and design of mask, and a user's head shape and wearing behavior, as these factors may cause aerosol leakage if a mask is poorly fitted. In addition, the use of air conditioners and the effects of wind can alter the distribution of aerosols. The effects of these factors can all be examined in future studies.

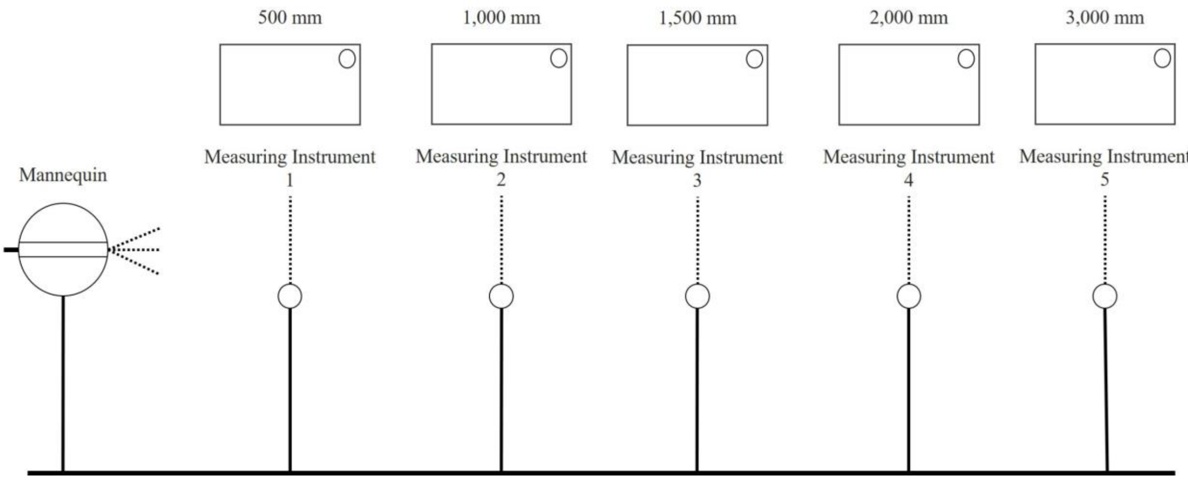

Fig. S1. Cough Aerosol Simulator (CAS).

This device was used to measure the distribution and mass concentration of micro-sized aerosols using OPC (11-D, Grimm, Germany) at locations 0.5, 1, 1.5, 2, and 3 m distant from the aerosol outlet. The inlet for sampling was parallel to the line of flow to ensure the best efficiency. The aerosol generation height was 1.6 m and the sampling height was 1.5 m, taking into account the slight downflow.

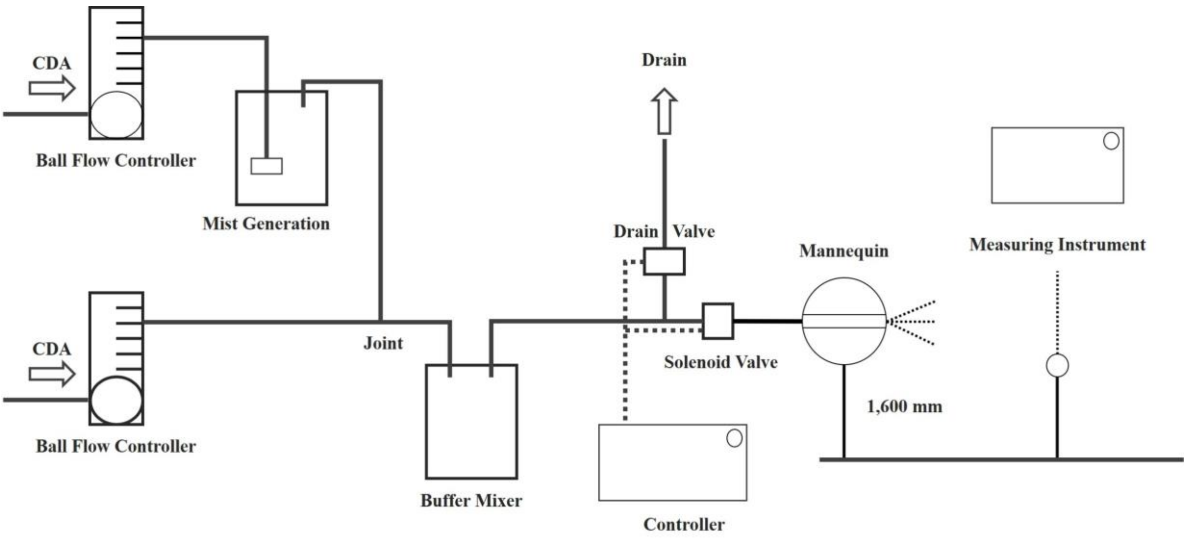

Fig. S2. Modified Cough Aerosol Simulator (CAS).

This device was designed to measure the distribution and mass concentration of nano-sized aerosols using ELPI (ELPI+, Dekati, Finland) at locations 0.5, 1, 1.5, 2, and 3 m distant from the aerosol outlet. The airflow for mist generation and coughing was 10 lpm and 220 lpm. The mannequin's solenoid valve (cough valve) was closed and the drain valve was open with mist generation air flow and without coughing airflow. During coughing, the drain valve was closed and the cough valve was open.

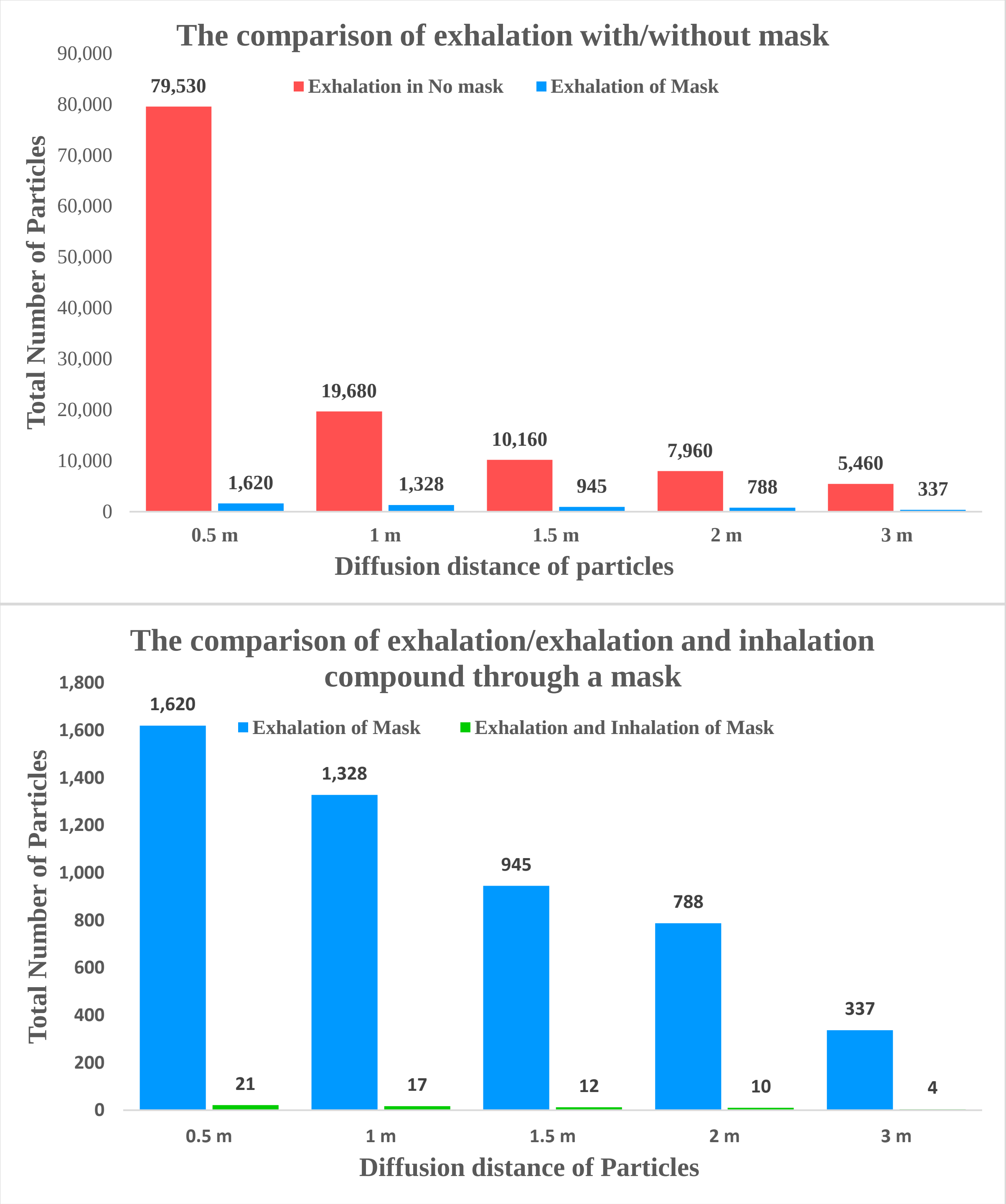

Fig. S3. Effect of mask-wearing during inhalation and exhalation on the spread of microsize particles.

The experiment showed that when the mask was not worn, approximately 10% of the particles in the solution representing cough aerosols spread farther than 2 m. In contrast, when the mask was worn most of these particles were blocked by the mask, and the most of the particles that passed through the mask were smaller than 576 nm, as shown in **Table S1.**

Table S1. Distance distribution for micrometer-scale particles with or without mask.

Classification	size	Distance				
		0.5 m	1 m	1.5 m	2 m	3 m
No mask	0.253 μm	16,160	6,170	3,910	2,710	1,660
	0.298 μm	13,970	4,150	2,550	1,950	1,100
	0.352 μm	9,060	1,950	1,000	800	450
	0.414 μm	11,220	1,950	750	600	550
	0.488 μm	11,730	2,110	700	650	550
	0.576 μm	10,440	2,050	850	850	750
	0.679 μm	6,950	1,300	400	400	400
	total	79,530	19,680	10,160	7,960	5,460
KF94 (exhalation)	0.253 μm	1,212	1,005	622	575	227
	0.298 μm	43	0	0	0	0
	0.352 μm	178	178	178	105	105
	0.414 μm	77	77	77	58	5
	0.488 μm	110	68	68	50	0
	0.576 μm	0	0	0	0	0
	0.679 μm	0	0	0	0	0
	total	1,620	1,328	945	788	337
KF94 (exhalation and inhalation)	0.253 μm	16	13	8	7	3
	0.298 μm	1	0	0	0	0
	0.352 μm	2	2	2	1	1
	0.414 μm	1	1	1	1	0
	0.488 μm	1	1	1	1	0
	0.576 μm	0	0	0	0	0
	0.679 μm	0	0	0	0	0
	total	21	17	12	10	4
Reduction in infection rate (exhalation)		97.96%	93.25%	90.70%	90.10%	93.83%
Reduction in infection rate (exhalation and inhalation)		**99.97%**	**99.91%**	**99.88%**	**99.87%**	**99.92%**

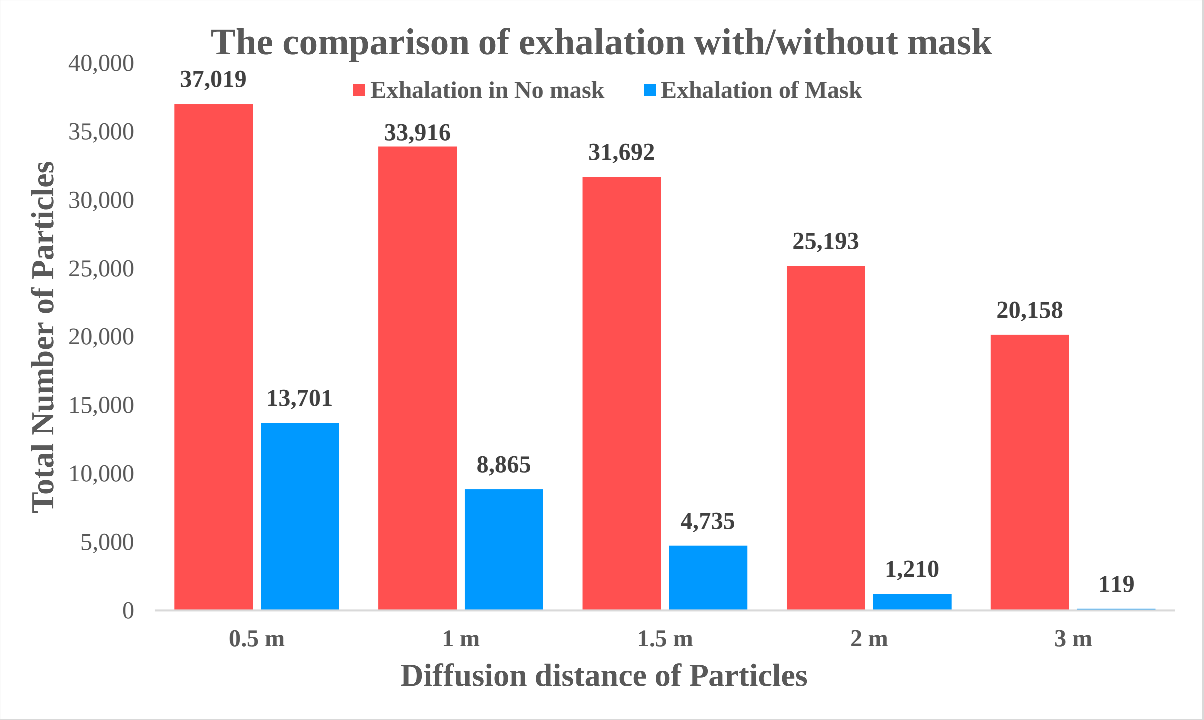

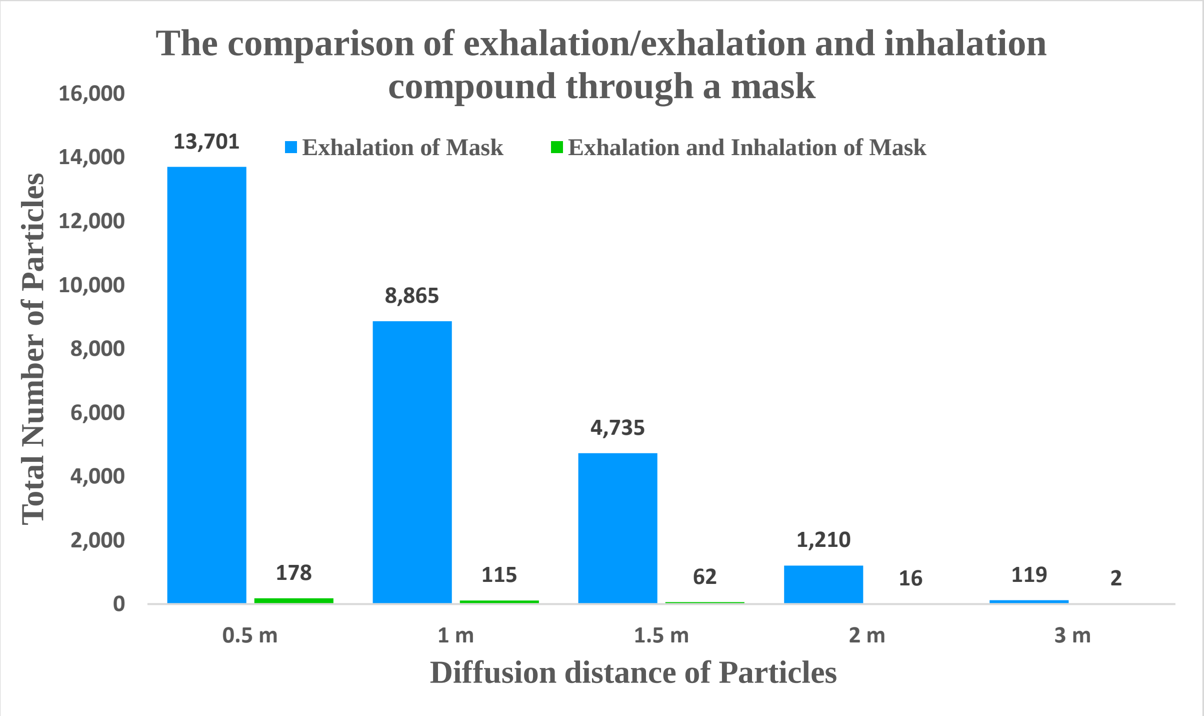

Fig. S4. Effect of mask-wearing during inhalation and exhalation on the spread of nanosize particles.

This experiment focused on the particle sizes mentioned in **Fig. S3** and **Table S1.** The results of the analysis indicated that mask-wearing blocked most particles 576 nm or larger in size, and that only a few particles smaller than 576 nm passed through the mask. This result proves that masks can block most particles in the solution, which represent cough aerosols. The number of viruses is likely to be proportional to the fluid volume of the particles. Particles 576 nm or larger in size, which exerted a significant volumetric effect, did not pass through the mask, and mask-wearing reduced both the number and volume of the particles reduced by more than 99.8%. Therefore, we assumed that the reduction in the infection rate is related to the reduction in the number of particles. However, on assessing the safety of travel in public transportation, we observed that the interiors of public transportation vehicles were not securely ventilated. Moreover, the degree of contact between passengers and the contact time were relatively high, which can increase the risk of infection. Therefore, the experiment was

conducted with a focus on nanosize particles that can pass through a mask and thereby pose an infection risk. The experiments showed that when a mask is not worn, virus particles can spread farther than 3 m and that wearing a mask limits this distance to less than 2 m, even if the particles pass through the mask. We determined that mandatory mask-wearing results in COVID-19–preventive effects similar to those resulting from maintaining 2-m physical distancing. We applied these experimental results to the diffusion model of a public transportation scenario.

Table S2. Distance distribution for nanometer-scale particles with or without mask.

Classification	size	Distance				
		0.5 m	1 m	1.5 m	2 m	3 m
No mask	0.010 µm	34,149	31,202	29,040	23,122	18,573
	0.021 µm	2,332	2,216	2,177	1,766	1,406
	0.040 µm	484	445	424	277	163
	0.071 µm	0	0	0	0	0
	0.121 µm	55	53	51	28	17
	0.198 µm	0	0	0	0	0
	0.312 µm	0	0	0	0	0
	total	37,019	33,916	31,692	25,193	20,158
KF94 (exhalation)	0.010 µm	11,879	7,786	4,166	1,053	88
	0.021 µm	1,258	785	431	119	26
	0.040 µm	342	183	95	30	4
	0.071 µm	112	43	11	0	0
	0.121 µm	91	55	27	7	1
	0.198 µm	18	13	5	2	0
	0.312 µm	0	0	0	0	0
	total	13,701	8,865	4,735	1,210	119
KF94 (exhalation and inhalation)	0.010 µm	154	101	54	14	1
	0.021 µm	16	10	6	2	0
	0.040 µm	4	2	1	0	0
	0.071 µm	1	1	0	0	0
	0.121 µm	1	1	0	0	0
	0.198 µm	0	0	0	0	0
	0.312 µm	0	0	0	0	0
	total	178	115	62	16	2
Reduction in infection rate (exhalation)		62.99%	73.86%	85.06%	95.20%	99.41%
Reduction in infection rate (exhalation and inhalation)		**99.52%**	**99.66%**	**99.81%**	**99.94%**	**99.99%**

2. Encounter Network analysis in Public Transportation

2.1 Construction trip chain using smart card data

In this study, we constructed real travelers' trip chains using smartcard data and household travel survey data. Smartcard data contain the encrypted identifier (ID) of the transit user, their location, their boarding time, and their time of disembarking, as shown in **Table S3**. To realize the encounter network for public transport journeys, we composed the trip chain of each user using their smartcard data ID-specific travel record. Furthermore, based on the configured trip chain, each user's time zone origin–destination (OD) pair and activity were tracked. Finally, we used household travel-survey data to construct the trip chains outside of the public transportation system. However, the data provide information about homes and workplaces based on zone units. Therefore, this study focused on encounters within the public transportation system along the total trip chain, as shown in **Fig. S5.**

Table S3. Sample of the smartcard data from Seoul applied in this study.

ID	Origin			Destination		
	Time	X	Y	Time	X	Y
1	08:50	127.1022	37.51395	09:05	127.056	37.50899
2	08:51	126.8234	37.49226	09:19	126.9264	37.51334

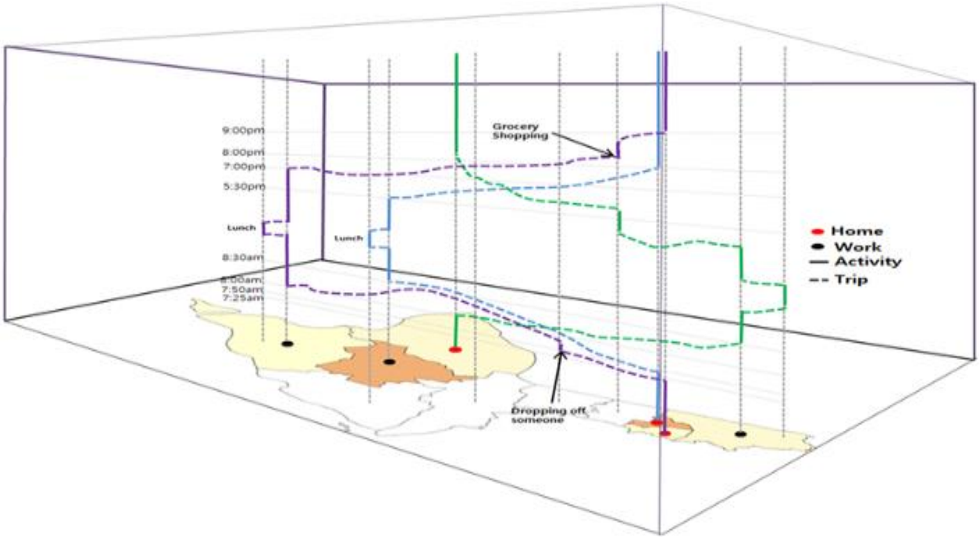

Fig. S5. Construction of trip chains using smart card data and household travel-survey data.

2.2 Public Transportation Trip Assignment using the agent based Model

In this study, we implemented the trip chain based on smart card data and performed traffic assignment under the agent based model. However, when using a smart card composed of only OD pairs, the movement path information of individual users is not provided. Hence, it is necessary to estimate the movement path of each individual with respect to time through the process of traffic assignment. Based on these traffic assignment steps, it is possible to implement an individual user's movement route with respect to time zone, congestion of each section, and encounter network. We performed the traffic assignment in the shortest route in public transportation by referring to the manual that explained as to how to use the multi-agent transport simulation MATSim *(24, 14)*. The study was conducted with MATSim, which is a transportation simulation focused on transportation planning *(25)*, and the user's shortest route estimation *(26)*. The shortest route-based traffic assignment model is as follows.

$$\begin{aligned}
C_{iv}(t) &= (\alpha_{iv} * t_{iv}(t))(1 + g(p_{oc}(t))) + \alpha_{vd} * l_{iv} + f_{iv} * l_{iv} \\
C_{bo}(t) &= \alpha_{wt} * t_{wt}(t) + c_{tr} \\
C_{al}(t) &= 0 \\
C_{wk}(t) &= \alpha_{wk} * t_{wk} + \alpha_{wd} * l_{wk} \\
C_{path}(t) &= \sum C_{iv}(t') + \sum C_{bo}(t') + \sum C_{al}(t') + \sum C_{tr}(t') - c_{tr}
\end{aligned} \tag{1}$$

where C_{path}: Total *cost of the path*

C_{iv}: *Cost of one in-vehicle link*

C_{bo}: *Cost of one boarding link*

C_{al}: *Cost of one alighting link*

C_{wk}: *Cost of one walking link*

α_{iv}: *Personalized cost per unit of time traveling in a vehicle*

α_{vd}: *Personalized cost per unit of distance traveling in a vehicle*

α_{wt}: *Personalized cost per unit of time waiting in a stop*

α_{wk}: *Personalized cost per unit of time walking*

α_{wd}: *Personalized cost per unit of distance walking*

c_{tr}: *Personalized cost for making a transfer*

f_{iv}: *Vehicle dependent fare rate with respect to distance traveled*

$t_{iv}(t)$: *In-vehicle travel time (from Stop-stop travel time structure)*

$t_{wt}(t)$: *Waiting time (from Stop-route waiting times structure)*

t_{wk}: *Walking time*

l_{iv}: *In-vehicle distance*

l_{wk}: *Walking distance*

$p_{oc}(t)$: *Occupancy level in the in-vehicle link (from Route-stop occupancy structure)*

$g(p)$: *Simplified function of how occupancy level increases the cost (Equation (2))*

$$g(p) = \begin{cases} 0 & if \quad p \le p_{sit} \\ r_{sta} * p + b_{sta} & if \quad p_{sit} < p < 1 \\ b_{full} & if \quad p = 1 \end{cases} \tag{2}$$

where, p_{sit}: *Occupancy level when no seats are available*

r_{sta}, b_{sta}: *Parameters denoting percentage increase in discomfort because of standing in a vehicle*

b_{full}: *Maximum percentage increase when the vehicle is full*

We set the congestion level for each section of the corresponding route with respect to time zone, as illustrated in **Table S4.** and **Fig. S6.** Subsequently, based on the constructed result, we implemented the encounter network to calculate the contact duration.

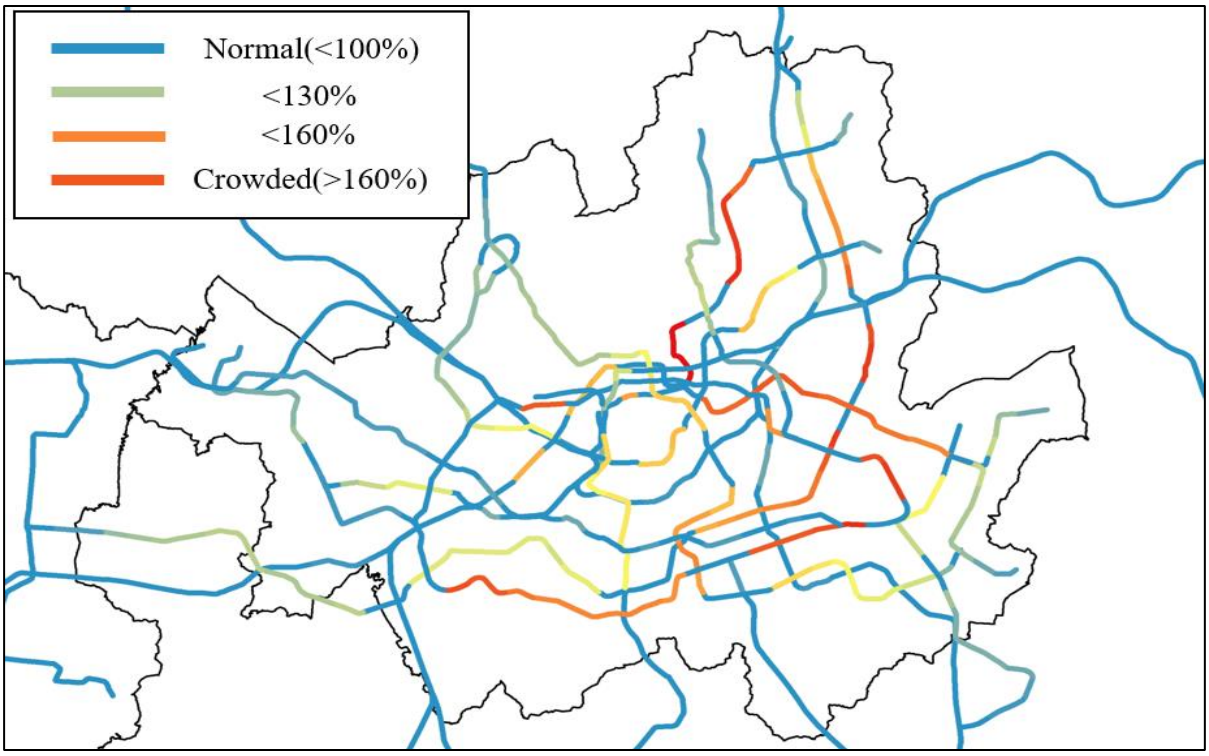

Fig. S6. Congestion map of the Seoul Metropolitan Subway Network.

We analyzed this congestion with respect to each section and reflected it in the infection rate of each section. Furthermore, **Fig. S6.** shows a map of congestion in the Seoul Metropolitan Subway Network via transit assignment using MATSim.

Table S4. Degree of Congestion in public transportation during peak hours.

Time	Line	Station to Station	Number of Cars	Degree of Congestion (estimated)	Degree of Congestion (real)
08:30	2	Bangbae-Seocheo	10	170.3	161.3
08:30	2	Sadang-Naksungdae	10	166.2	154.2
08:00	4	Naksungdae-Seoul National Univ	10	153.6	152.9
08:30	2	Dongdaemon-Hyehwa	10	145.9	151.4
08:30	2	Seoul National Univ-Bongchun	10	135.3	128.2
08:30	4	Gil Eum-Mia Intersection	10	124.7	122.3
08:00	2	Jamsil Saenae-Jamsil	10	115.3	100.8
08:00	2	Daelim–Guro Digital Complex	10	105.6	95.8
08:00	2	Euljiro 3-ga-Euljiro 4-ga	10	95.5	80.8
07:30	2	Hongdae Entrance-Sinchon	10	85.1	77.4
08:30	5	Gunja-Janganpyeong	10	75.5	74.0
07:30	2	Nakseongdae-Sadang	10	65.6	73.2

In this study, congestion indicators were used, and the basic data for calculating them were large direct current electric vehicles operating in Seoul, which used the standard standards for urban railway vehicles. The passenger quota is also defined as having an area of $0.35m^2$ per person, with 160 passengers per vehicle, with a 100% congestion. The more in this, the lower the level of service, the higher the congestion, and the lower the average distance between each agent, which increases the probability of infection of respiratory diseases. When there is about 170% congestion, there is virtually no empty space in the vehicle, where each agent is closely related to each other in terms of possible infection. In this study, the area per person was assumed to be circle and converted into the distance between individuals. As a result, the 100% congestion-based agent is found to be about 1.3m apart, and the 160% congestion-based distance is 1m, except for the area occupied by the human body, there is virtually no empty space. We divided level of service in public transportation according to congestion level *(27).* Next, we combined the average distance between the individual agents and the changes in the particle distribution according to the distance. This combined value is reflected by the change (C) in the probability of an individual being infected. We can express the ratio of the number of particles to the distance, which has nonlinear characteristics, as a linear regression function (**Fig. S7**), where x is the distance and y is the number of particles observed over this distance. The coefficient, y-intercept, and R^2 are −7,011.2, 40,813, and 0.9476, respectively. As the minimum distance between the individual agents is 0.51 m, we also set the minimum observational diffusion value to 0.5 m. Ratio C is set to 1 at a minimum distance of 0.5 m between agents; as the average distance increases, Ratio C decreases according to the linear regression function as follows.

$$\text{Ratio } C = \frac{number\ of\ particles\ based\ on\ each\ distancing\ agent}{number\ of\ particles\ based\ on\ a\ distance\ of\ 0.5m}$$

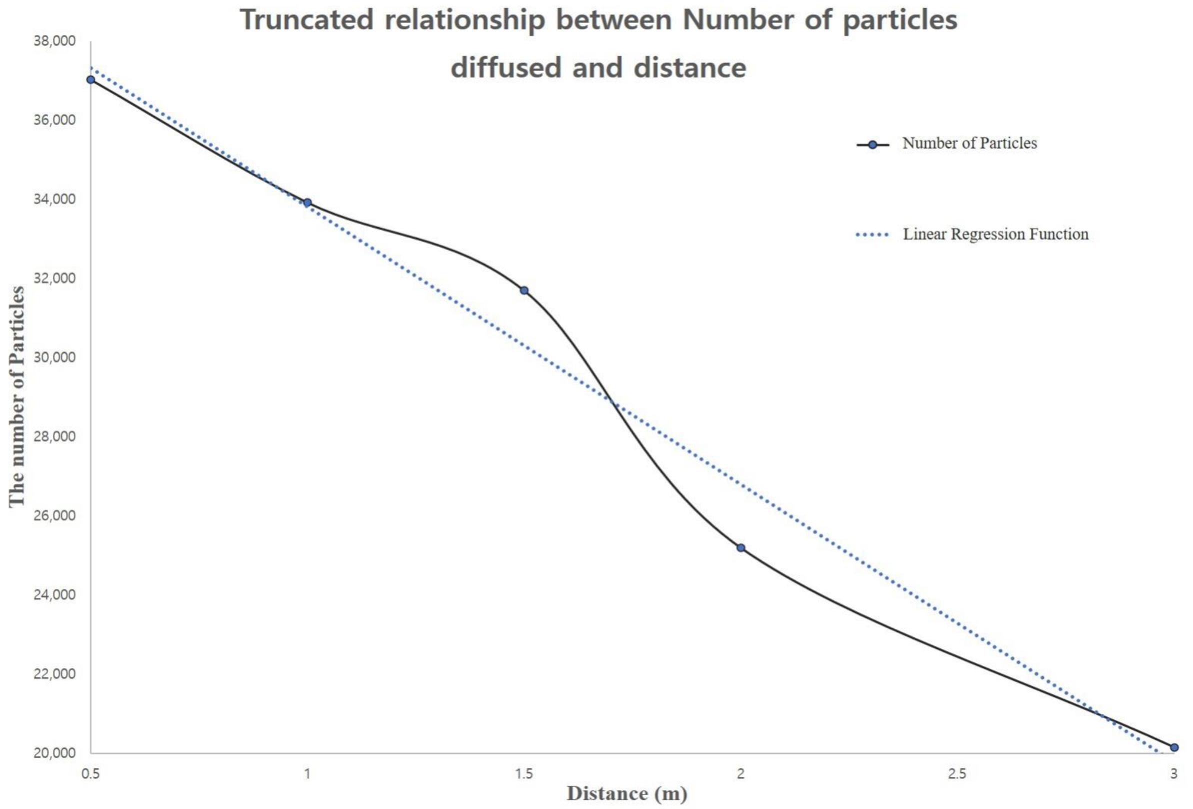

Fig. S7. Reduction in Ratio $\boldsymbol{C}$ with distance.

Table S5. Area occupied by each agent and average distance between agents depending on the degree of congestion.

Num. of Agents in a Compartment	Congestion (%)	Level of Service	Occupied area of each agent (m^2)	Average Distance of agents (m)	Ratio $\boldsymbol{C}$
272	170	F	0.21	0.51	1.00
256	160	E	0.22	0.53	1.00
240	150		0.23	0.55	0.99
224	140		0.25	0.56	0.99
208	130		0.27	0.59	0.98
192	120		0.29	0.61	0.98
176	110	D	0.32	0.64	0.98
160	100		0.35	0.67	0.97
144	90		0.39	0.70	0.96
128	80		0.44	0.75	0.95
112	70		0.50	0.80	0.95
96	60		0.58	0.86	0.93
80	50	C	0.70	0.94	0.92
64	40		0.88	1.06	0.90
48	30	B	1.17	1.22	0.87
32	20	A	1.75	1.49	0.82
16	10		3.50	2.11	0.70

2.3 Familiar Stranger group in Encounter Network

In the public transportation passenger encounter pattern studied by Sun and colleagues *(6)* through an encounter network, each node represents a passenger and each edge represents an undirected graph that represents a pair of passengers staying in the same vehicle. Smart card data, including passenger commuting time, location, and corresponding bus ID, were analyzed to form a network. The network was expanded by Sun and colleagues *(6)*, and they proposed a time-varying weighted Public Transportation Encounter Network (PEN) that can model an infectious process. Specifically, PEN is an ideal tool for investigating the spread of infectious diseases through public transportation services because it provides passengers information on direct contact with infected cases.
First, we divide the entire study period by an even time interval $t = 1, \ldots, T$ of length τ. With respect to the weighted graph $G_t(\mathcal{N}, \mathcal{E}_t, \mathcal{W}_t)$, $\mathcal{N} = \{i : i = 1, \ldots, N\}$, the node set is used, where each node represents an individual, and N is the total number of passengers. $\mathcal{E}_{\mathrm{t}}$ denotes the edge set and $\mathcal{W}_{\mathrm{t}}$ denotes the weight set. The edge between i and j ($i, j \in \mathcal{N}$) denoted by e_{ij}^t remained in the same vehicle when considering all i and j values during the time interval t. The weight of e_{ij}^t expressed as w_{ij}^t is $w_{ij}^t = \frac{d_{ij}^t}{\tau}$. Furthermore, d_{ij}^t denotes the duration of i and j staying in the same vehicle for an interval t. By definition, $0 \leq w_{ij}^t \leq 1$. Weights are used to capture the fact that epidemic transmission is related to the duration of contact.

2.4 Social-activity contact network

There are various other situations, including public transportation, wherein passengers can come in contact with each other. These situations include social activities (SA) where passengers are heterogeneously distributed in terms of space. However, to capture the actual connectivity of passengers during daily activities, rich datasets (e.g., mobile data, GPS data) are required. In this study, we made the following assumptions to build the SA contact network.

- Global interaction: Passengers can interact with other individuals in the system with a uniform probability θ_g during time interval t.
- Local interaction: Passengers of the same origin or destination of a PT trip can interact with each other with a uniform probability during time interval t. $\theta_l > \theta_g$ holds because local interaction is stronger than global interaction.

In global interaction, it is assumed that the contact time of all connected individuals, when there is no local contact with PT, is τ for a specific time interval. Conversely, the contact time should be reduced by PT and local contact duration (CD) at the time interval. In local interaction, the contact time calculation is as follows. There is a passenger i whose PT trip sequence is $\{(O_{t_1}^i, D_{t_2}^i), (O_{t_3}^i, D_{t_4}^i)\}$, where t_k denotes the time when the passenger boards the vehicle. Furthermore, $O_{t_k}^i$ *and* $D_{t_{k'}}^i$ denote the trip origin and destination, respectively. A trip sequence is defined as a sequence of consecutive PT trips, where all adjacent trip pairs exhibit an interval of less than 24 h (e.g., $t_3 - t_2 < 24h$). Additionally, $D_{t_2}^i \neq O_{t_3}^i$ may hold because the passenger may not be able to stay in the same place between two consecutive trips. It is also assumed that from t_2 to t_3 the passenger spends half of the activity time at $D_{t_2}^i$ and half of the activity time at $O_{t_3}^i$.
The trip sequence of passenger j is $(O_{t_1}^i, D_{t_2}^i), (O_{t_3}^i, D_{t_4}^i)$, with $D_{t'_2}^i = O_{t_3}^i$. There is no overlapping time between the intervals $[t_2, t_3]$ and $[t'_2, t'_3]$. The i and j passengers stay at the same location $D_{t'_2}^i = O_{t_3}^i$. Hence, they may have a local contact (the possibility of local contact is indicated by θ_l). It is assumed that the passenger spends half of the time of an activity at a specific origin or destination. If they have a local contact, then the CD between passengers i and j is half the time of overlapping between interval $[t_2, t_3]$ and interval $[t'_2, t'_3]$. This calculation provides the total CD of i and j at the local interaction level. For example, if $t_2 < t'_2 < t_3 < t'_3$, then the total local CD between i and j is $\frac{1}{2}(t_3 - t'_2)$. Similarly for PEN, the entire local CD can be mapped to each time interval. For example, t^* is the time boundary for time interval t and time interval t + 1, and $t^* - \tau < t'_2 < t^* < t_3 < t^* + \tau$ to be. We denote the local CD between i and j for time interval t as $\tilde{d}_{ij}^{l,t}$ ($0 \leq \tilde{d}_{ij}^{l,t} \leq \tau$). Subsequently, $\tilde{d}_{ij}^{l,t} = t^* - t'_2$ and $\tilde{d}_{ij}^{l,t+1} = t_3 - t^*$.
The SA contact network is $\tilde{G}_t(\mathcal{N}, \tilde{\mathcal{E}}_{g,t}, \tilde{\mathcal{E}}_{l,t}, \tilde{\mathcal{W}}_t^g, \tilde{\mathcal{W}}_t^l)$, where $\tilde{\mathcal{E}}_{g,t}$ denotes the edge set of global interaction and $\tilde{\mathcal{E}}_{\mathrm{l,t}}$ denotes the edge set of local interaction. The edge of the global interaction between all i and j denoted by $\tilde{e}_{ij}^{g,t}$ exists as a probability θ_g for all $i, j \in \mathcal{N}$. When i and j share the same PT trip origin or destination during time interval t, the edge of local interaction between i and j ($\tilde{e}_{ij}^{l,t}$) exists with θ_l probability. Furthermore, $\tilde{\mathcal{W}}_t^g$ and $\tilde{\mathcal{W}}_t^l$ denote the weights set on the global and local interaction edges, respectively.
We developed an encounter network on the public transportation network based on the OD pair of smart card data

wherein the traffic assignment was completed. Furthermore, we built an actual public transportation network based on the operational routes and timetables with respect to time zone with the goal of realizing accurate public transportation users. During the construction of encounter network, we analyzed the users, including existing occupants, passengers, and future occupants, who met for a minimum contact time of 10 min. Subsequently, a familiar stranger group is created based on the individual users and estimated encounter network. Hence, the standard of encounter is as follows.

$$T_{R_j} < T_{R_i} \ \& \ T_{R_i} < T_{A_j} = Encounter \quad (3)$$
$$T_{R_i} < T_{R_j} \ \& \ T_{R_j} < T_{A_i} = Encounter \quad (4)$$
$$T_{A_j} < T_{R_i} = No\ Encounter \quad (5)$$
$$T_{A_i} < T_{R_j} = No\ Encounter \quad (6)$$

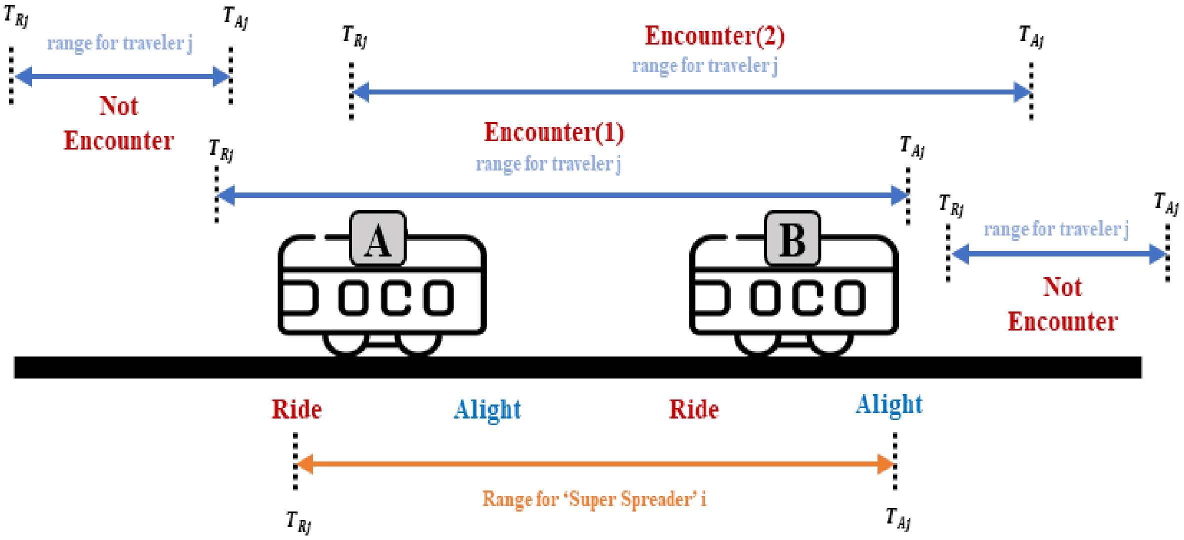

Fig. S8. Encounter Network analysis.

3. Path analysis of Exposures

3.1 Infectious Pathways

In South Korea, the Centers for Disease Control and Prevention (CDCP) provides the actual path of infection through epidemiological investigations. This data is provided in the form of OD pair for each time zone, similar to that in smart card data, from 00 days before the actual infected person receives confirmation of infection until the time when they receive confirmation or return to hospital or are in quarantine. In this study, we exploited data of real infected individuals available from the epidemiological survey data. As in the previous methodology, we assigned the OD pair for the movement of the infected person through the traffic assignment based on the shortest path route selection. Furthermore, the CDCP released data on epidemiological investigations of individuals who received COVID-19 confirmation on February 25, 2020. In the study, we determined that an individual used the public transportation from Yeouinaru Station to Songjeong Station on February 22, 2020 by using the smart card data and data on real infected individuals. Hence, in the study, we estimated and applied the intermediate stage movement (time zone for each section, etc.) during the movement, as shown in **Table S6.**

Table S6. Epidemiological Survey Data on the Movement of Public Transportation by Actual Infectious (2020).

Date	Time	Origin Station	Destination Station	Date of infection
2020.02.22	11:00	Yeoinaru	Songjeong	2020.02.25
2020.02.25	08:00	Gangnam	Gyeongbokgung	2020.02.29
2020.02.25	10:30	Suseo	Kuangnaru	2020.02.18
2020.01.31	10:30	Sungsin Univ	Sangwangsipli	2020.01.24
:	:	:	:	:

3.2 Applying an SEIR structure

We performed infection diffusion pathway analysis based on the analyzed individual encounter network. In a previous study, an SEIR structure was applied as an infection diffusion path. The SEIR model generally represents the degree of spread of respiratory infections and divides the population into four stages. In this study, an SEIR structure was applied according to the contact duration (CD) calculated from the PEN in the public transportation network.

We first classified the population into four classes according to the stage of the disease *(28, 29, 30)*:

- Susceptible (denoted by S, those who can contract the infection)
- Exposed (E, those who are infected with the disease, but they are unable to spread or can only spread the virus with low probability)
- Infectious (I, those who are infected and are contagious)
- Recovered (R, those who are removed from the spread, either because they have recovered from the disease with an immune process or they have died).

By definition, $\mathcal{N} = S \cup E \cup I \cup R$, and $\mathcal{N}$ is the set of the entire population. The SEIR model can explain as to how an individual passes through each compartment of the model. The infection rate β controls the proliferation rate and is related to the probability of disease spread between a susceptible (S) and exposed individual (E). The incubation rate, γ, is the rate at which an exposed individual (E) becomes infectious (I). The removal rate, μ, is the sum of recovery and mortality due to COVID-19. In the SEIR model, it is generally assumed that given the immunity obtained, the recovered individual will not be infected again. This study focuses on the early stages of the epidemic process, where the effect of external factors on $\mathcal{N}$ (e.g., birth and natural history) is not considered. In the case of the epidemic process model, individuals are concerned about the steady state, epidemic threshold, and reproduction number. According to Pastor–Satorras and colleagues *(31)*, the number of individuals infected in the SEIR model tends to be zero even after prolonged periods (see **Fig. S11**) (*31, 32*). This can be seen in the SEIR structure where only state R is the only absorbing state (e.g., no transitions out). The default reproduction number represented by R_o is defined as the average number of secondary infections due to primary cases introduced into the fully susceptible population *(28)*. In the standard SEIR model, $R_o = \frac{\beta}{\mu}$. In many cases, the pandemic threshold is defined based on the value of R_o. When $R_o < 1$, the number of infectious individuals tends to decline exponentially, which indicates no epidemic. However, if $R_o > 1$, the number of infectious individuals may increase exponentially due to the occurrence of an epidemic, as shown in **Fig. S9.** and it is analyzed using the SEIR model, which is widely used for modeling infectious diseases. The movement between the four groups is considered according to parameters such as the transmission rate, recovery rate, average infection period, and incubation period of infectious diseases. The relevant parameter is calculated and applied to the domestic situation based on statistical data from the Korea Centers for Disease Control and Prevention. Furthermore, January 21, when the first case was confirmed, is set as the start date of the analysis, as shown in **Table S7.**

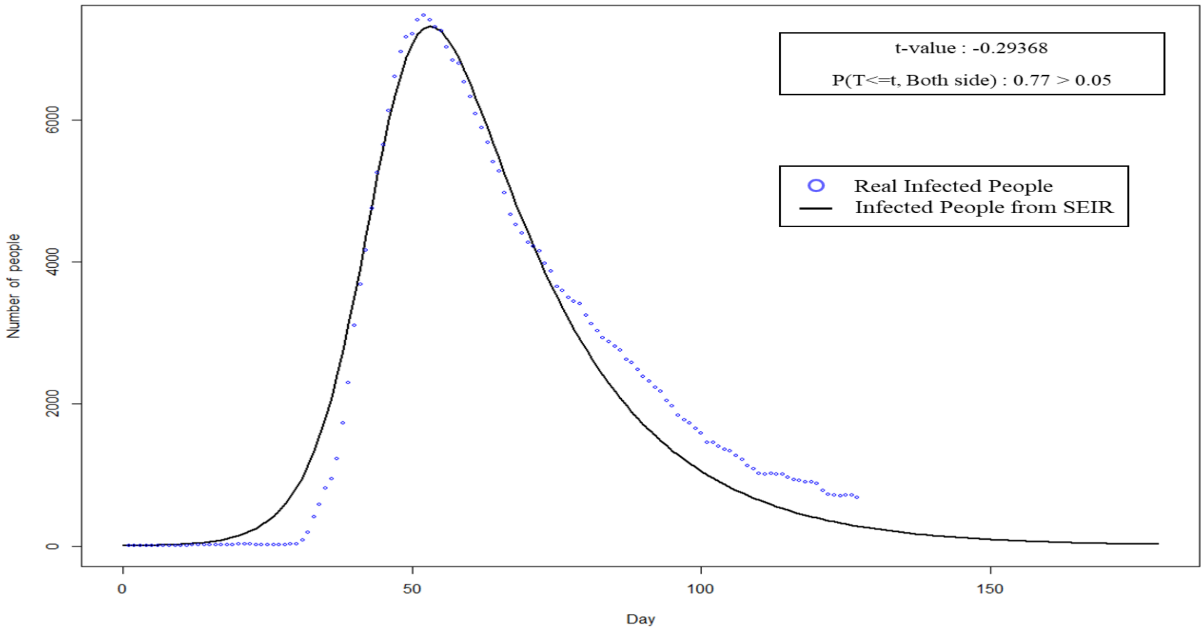

Fig. S9. Number of individuals in different classes in South Korea's COVID-19 ($\mathbf{R_0}$>1) epidemic.

Table S7. Exposure of infection based on the degree of contact duration under PTEN.

Day	Susceptible (%)	Exposed (%)	Infection (%)
1	100.0%	0.0%	0.0%
4	99.9%	0.1%	0.0%
10	99.7%	0.2%	0.0%
30	91.1%	6.9%	0.6%
60	3.7%	41.6%	5.6%
100	0.3%	6.4%	0.9%
180	0.5%	0.1%	0.0%

The typical model of an epidemic can be divided into two categories: individual-based and degree-based approaches. The individual-based approach models epidemic transmission at the individual level, and the degree-based approach captures the infection process at the group level. Each group contains a set of nodes (individual) of the same level. In the PEN, we can characterize the behavior of human interaction at the individual level. Therefore, individual-based framework was chosen in this study. The Seoul Metropolitan Subway is the world's fourth largest subways after in Beijing, Shanghai, and Guangzhou in China. The length of a single compartment is 20 m, thus, the total length of a train is usually 160 m to 200 m, with 8 to 10 compartments, respectively. In this large subway, it is possible for passengers to avoid contact with each other even if they board the same subway at the same time. Therefore, we defined the set of compartments with individual passengers on board to build an individual-based framework. Because it was difficult to confirm which compartment the actual individual agent was on, we assumed that the magnitude containing the contacted passenger was inversely proportional to the number of compartments on the subway line.

In an actual public transportation setting, users tag their smartcards at an entrance and move further ahead to board a subway. We used artificial intelligence techniques composed of a many-to-many type of deep neural network to track the compartments containing boarded agents according to the following procedure. First, according to the constructed trip chain, we tracked the agent's path from outside to inside the subway, and to the entrance where the smartcard was tagged. This was possible because we were able to obtain records of the first origin, destination, and public transportation usage of agents. Second, we defined the locations where the smartcard was tagged in the subway station, and the location of the nearest stairs and elevators, and thus predicted which compartment was selected after the smartcard was tagged. Third, we verified the actual number of passengers according to the weight of each compartment of the subway in Busan using the root-mean-square error (RMSE). In the Busan Metro system, a load-compensating sensor is installed in each compartment, and the data from this sensor enabled us to determine the number of passengers per compartment, as shown in **Fig. S10.**

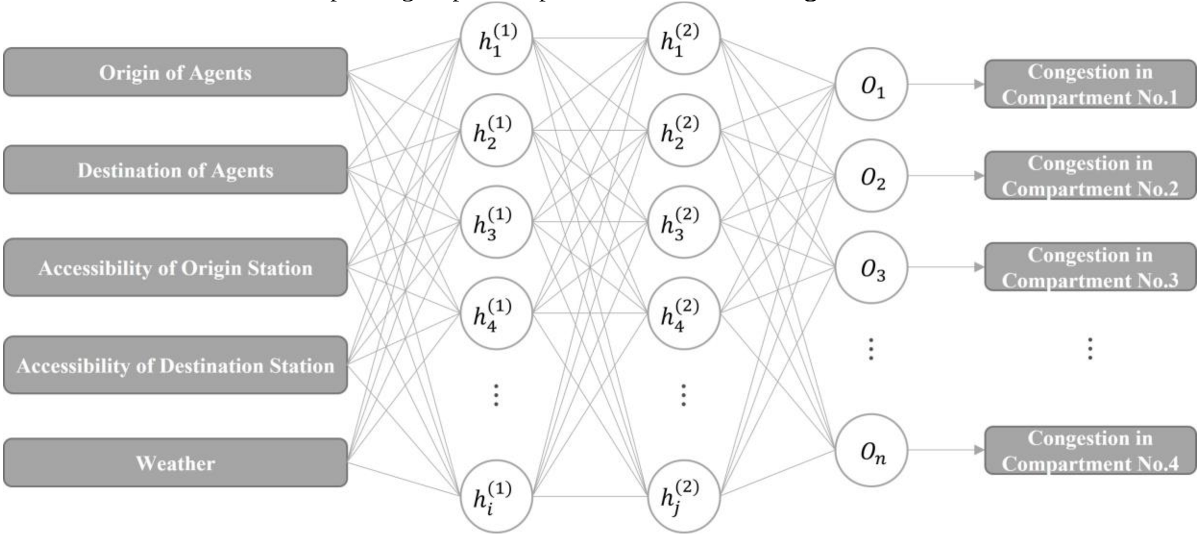

Fig. S10. Tracking the compartments containing boarded agents using artificial intelligence techniques.

However, the parameters obtained from the Busan Metro cannot be generalized to the Seoul Metropolitan Subway, which is the focus of this paper. We expect the predictions based on real data to be applicable to our current study.

We describe whether the individuals i are in classes S, I, E, and R at the time interval t with the Bernoulli random variables $S_{i,t}, I_{i,t}, E_{i,t}$, and $R_{i,t}$. By definition, $S_{i,t} + I_{i,t} + E_{i,t} + R_{i,t} = 1$ for all i and t. Let $\mathbb{P}(X_{i,t} = 1) = p_{i,t}^{X}$, where $X \in \{S, I, E, R\}$ and $\sum_{x} p_{i,t}^{x} = 1$. Given that the contact network was defined as discrete time, the epidemic process of the SEIR model can be described as a discrete Markov process with a certain probability of transition. To match the epidemiological properties of COVID-19, it is assumed that exposed individuals can infect others based on recent findings *(33)*, which may not be common in SEIR models.

If i and j come into contact with each other during the entire time interval (by PT or SA), then the probability of an individual $i \in S$ to be infected by an infected person $j \in I$ at time interval t is termed ζ_I. Given that the actual transmission probability is related to the duration of the interaction, the actual probability of i infected with $j(\zeta_{i,j,t}^{I})$ is as follows:

$$\zeta_{i,j,t}^{I} = a_{ij}^{t} \cdot h(w_{ij}^{t}, \zeta_I) \cdot C + \tilde{a}_{ij}^{l,t} \cdot h(\widetilde{w}_{ij}^{l,t}, \zeta_I) + \tilde{a}_{ij}^{g,t} \cdot h(\widetilde{w}_{ij}^{g,t}, \zeta_I) \quad \forall_i \in S, j \in I. \tag{7}$$

where $h(\cdot, \cdot)$ denotes a function that describes the actual transmission probability for a CD. It can be in the form of a survival function (e.g., exponential, Weibull) or a linear function (i.e., $h(\omega, \beta) = \omega\beta$ used in case studies). While $a_{ij}^{t}(\tilde{a}_{ij}^{l,t}, \tilde{a}_{ij}^{g,t})$ is a variable that indicates whether $e_{ij}^{t}(\tilde{e}_{ij}^{l,t}, \tilde{e}_{ij}^{g,t})$ exists, a_{ij}^{t} is a known constant. However, $\tilde{e}_{ij}^{l,t}$ and $\tilde{e}_{ij}^{g,t}$ are arbitrary variables obtained using the Bernoulli distribution: $\tilde{a}_{ij}^{l,t} \sim B(L_{ij}^{t}\theta_l)$ *and* $\tilde{a}_{ij}^{g,t} \sim (\theta_g)$. $L_{ij}^{t} = 1$ if i and j share the same origin or destination at time interval t, otherwise L_{ij}^{t}=0. Furthermore, the function C reflects the linear average distance of individual $i \in S$ and is calculated based on the level of public transport congestion. The reduction in infection probability with average distance was determined as conservatively as possible; that is, we considered the effect of distance based on the aerosol diffusion distance when no mask was worn. In the no-mask experiment (**Table S1**), the average distance between agents was considered to be 0.51 m under the highest level of congestion (170%). We then applied ratio C to the number of particles, which decreased as the average distance between individual agents increased. Thus, the following holds.

$$\zeta_{i,j,t}^{I} = (a_{ij}^{t} \cdot h(w_{ij}^{t}, \zeta_I) \cdot C + L_{ij}^{t}\theta_l \cdot h(\widetilde{w}_{ij}^{l,t}, \zeta_I) + \theta_l \cdot h(\widetilde{w}_{ij}^{g,t}, \zeta_I)) \quad \forall_i \in S, j \in I. \tag{8}$$

Similarly, we define ζ_E as the probability of $i \in S$ to be infected by the exposed individual $j \in E$ at time interval t if i and j are in contact with each other for the entire time interval ($\zeta_E \ll \zeta_I$). The actual transmission probability considering the duration of the interaction is as follows:

$$\zeta_{i,j,t}^{E} = a_{ij}^{t} \cdot h(w_{ij}^{t}, \zeta_E) \cdot C + L_{ij}^{t}\theta_l \cdot h(\widetilde{w}_{ij}^{l,t}, \zeta_E) + \theta_l \cdot h(\widetilde{w}_{ij}^{g,t}, \zeta_E) \quad \forall_i \in S, j \in E. \tag{9}$$

We assume that if {i} and {j} are in contact, then the probability of transmission depends only on the CD. The average distance between individual agents was used to calculate the change in transmission probability due to changes in the spatial distribution. This average distance was calculated based on the level of congestion in public transport, which was determined on the basis of the number of passengers on board and the actual area of a public transportation vehicle plying in Seoul. Specifically, γ denotes the probability of $E \rightarrow I$, which is not network related, and μ denotes the probability of $I \rightarrow R$ and $\mu = \mu_r + \mu_d$. Furthermore, μ_r and μ_d are the probabilities that infected individuals will be treated and die, respectively, and neither of the probabilities are related to the network. Thus, using the notation and epidemic transmission mechanism, the following system equations can be written as follows:

$$p_{i,t+1}^{S} = p_{i,t}^{S} - \sum_{j \in \mathcal{N}} \zeta_{i,j,t}^{E} \mathbb{P}(S_{i,t} = 1,\ E_{i,t} = 1) - \sum_{j \in \mathcal{N}} \zeta_{i,j,t}^{I} \mathbb{P}(S_{i,t} = 1,\ I_{i,t} = 1) \tag{10}$$
$$p_{i,t+1}^{E} = p_{i,t}^{E} + \sum_{j \in \mathcal{N}} \zeta_{i,j,t}^{I} \mathbb{P}(S_{i,t} = 1,\ I_{i,t} = 1) + \sum_{j \in \mathcal{N}} \zeta_{i,j,t}^{I} \mathbb{P}(S_{i,t} = 1,\ I_{i,t} = 1) - p_{i,t}^{E}\gamma \tag{11}$$
$$p_{i,t+1}^{I} = p_{i,t}^{I} - p_{i,t}^{I}(\mu_r + \mu_d) + p_{i,t}^{E}\gamma \tag{12}$$
$$p_{i,t+1}^{R} = p_{i,t}^{R} - p_{i,t}^{I}(\mu_r + \mu_d) \tag{13}$$

The calculation of $\mathbb{P}(S_{i,t} = 1, X_{j,t} = 1)$ requires a common distribution of $S_{i,t}$ and $X_{j,t}$ that are not typically available. According to the individual-based mean field approximation, it can be assumed that the states of the neighbors are independent *(34, 35, 36)*.

$$\mathbb{P}(S_{i,t} = 1, E_{j,t} = 1) = p_{i,t}^{S} p_{j,t}^{E} \tag{14}$$

$$\mathbb{P}(S_{i,t} = 1, I_{j,t} = 1) = p_{i,t}^{S} p_{j,t}^{I} \quad (15)$$

Therefore, we substituted Eqs. 14 and 15 into Eqs. 10 and 11 to obtain a new group of system equations that can be solved.

The research analyzed the encounter network with respect to time zone based on smart card data. In the case of public transportation, the characteristics of weekdays, weekends, and peaks are distinct. Hence, we performed an analysis by classifying the encounter network with respect to weekdays, weekend, and time zone into degrees and contact durations. We assumed an interval of 1 h. In the trend line in the graph above, a quadratic polynomial is selected. The encounter network is determined by the traffic volume for each section of the time zone and the OD pair of individual traffic, as shown in **Fig. S11. and Fig. S12.**

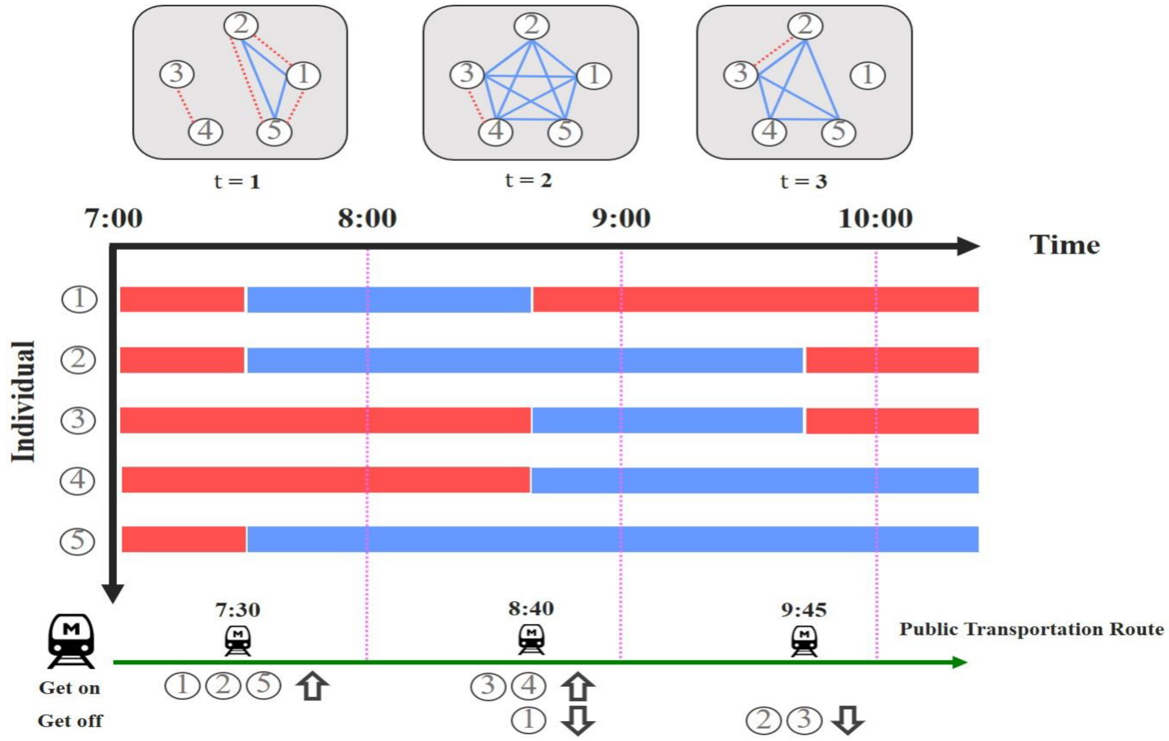

Fig. S11. Encounter Network Tracking of a five-passenger system.

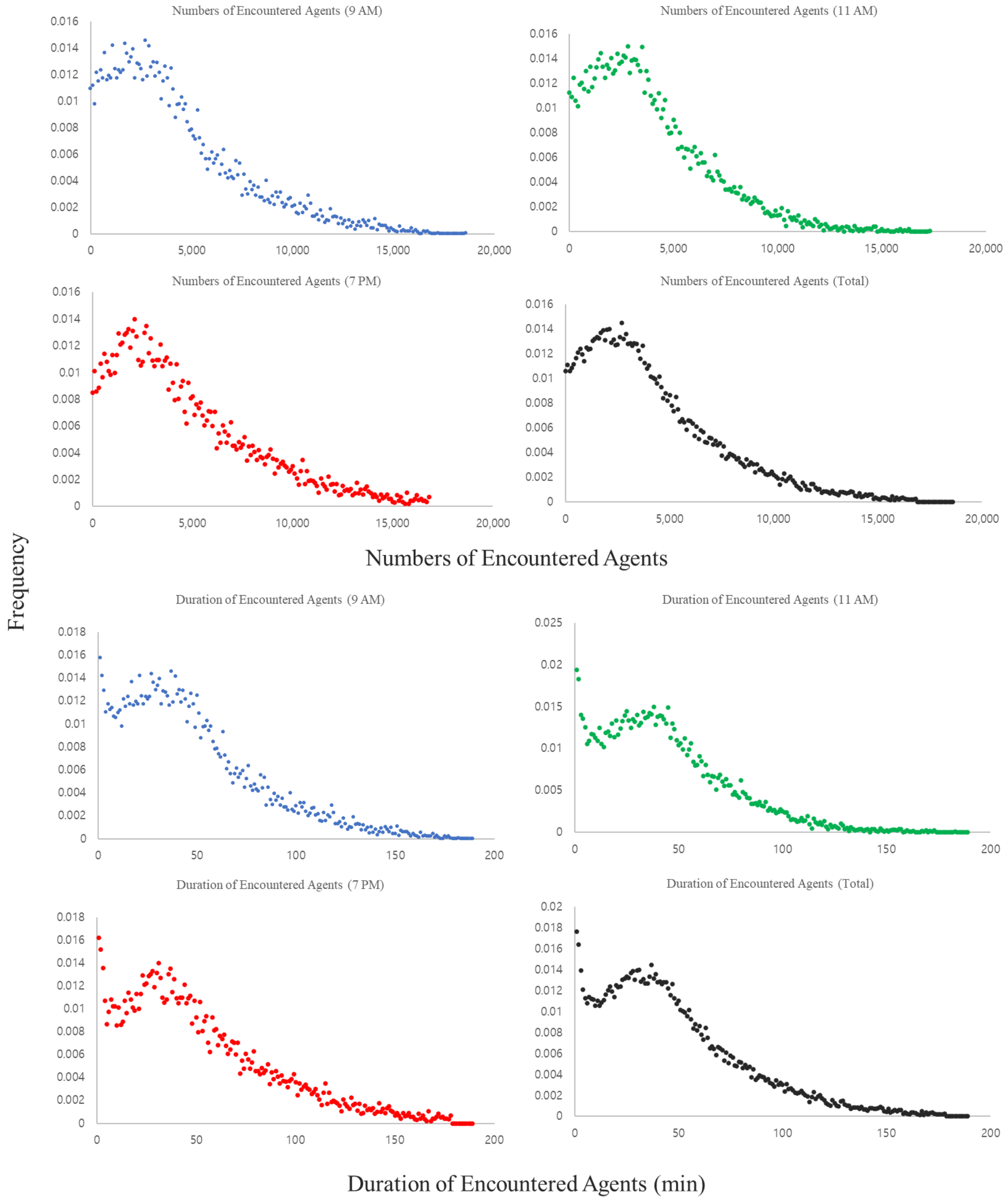

Fig. S12. Changes in the number and duration of encounters between agents with time.

Table S8. Numbers and durations of encounters between agents at different time.

Mean	7:00 AM	11:00 AM	7:00 PM	Days
Number of Encounters	4,177	3,793	4,626	4,199
Duration of Encounters (min)	47.22	43.30	51.45	47.31

3.3 Mobility patterns in Seoul Metropolitan subway

Seoul is one of the most public transportation friendly cities in the world. With the introduction of various demand management policies, more than 70% of people in Seoul use public transport (*37*). We validated the impact of the Encounter Network by including cases of real infected people who were using public transportation. The assignment of the environment in which the actual infected person used public transport was made using the activity-based model of the actual movement records of real infected people described in section 2.

First, we analyzed the high-demand routes in the encounter network, which varied over time, for specific validation. We tracked the actual infected person from the moment they boarded the compartment to the time of first encounter up to the fourth encounter. First, Line 5 was analyzed at 11:00 a.m. on February 22, 2020 (weekend), when the number of actual infected people was low, and the results are shown in the left-hand panels of **Fig. S13.** Second, Line 7 was analyzed at 9:00 a.m. (peak) on February 28, 2020 (week), when actual infected people used the line, and the results are shown in the middle panels of the figure. Third, Line 2 was analyzed at 9 p.m. on February 26, 2020 (week), when many infected people used the line, and the results are shown in the right-hand panels. Line 2 had the highest demand, as it passes through all of the core areas of Seoul, and even though it was a non-peak hour, the highest number of encounters occurred on this line, as shown in **Fig. S13., Fig. S14. and Movie S1 to S3.**

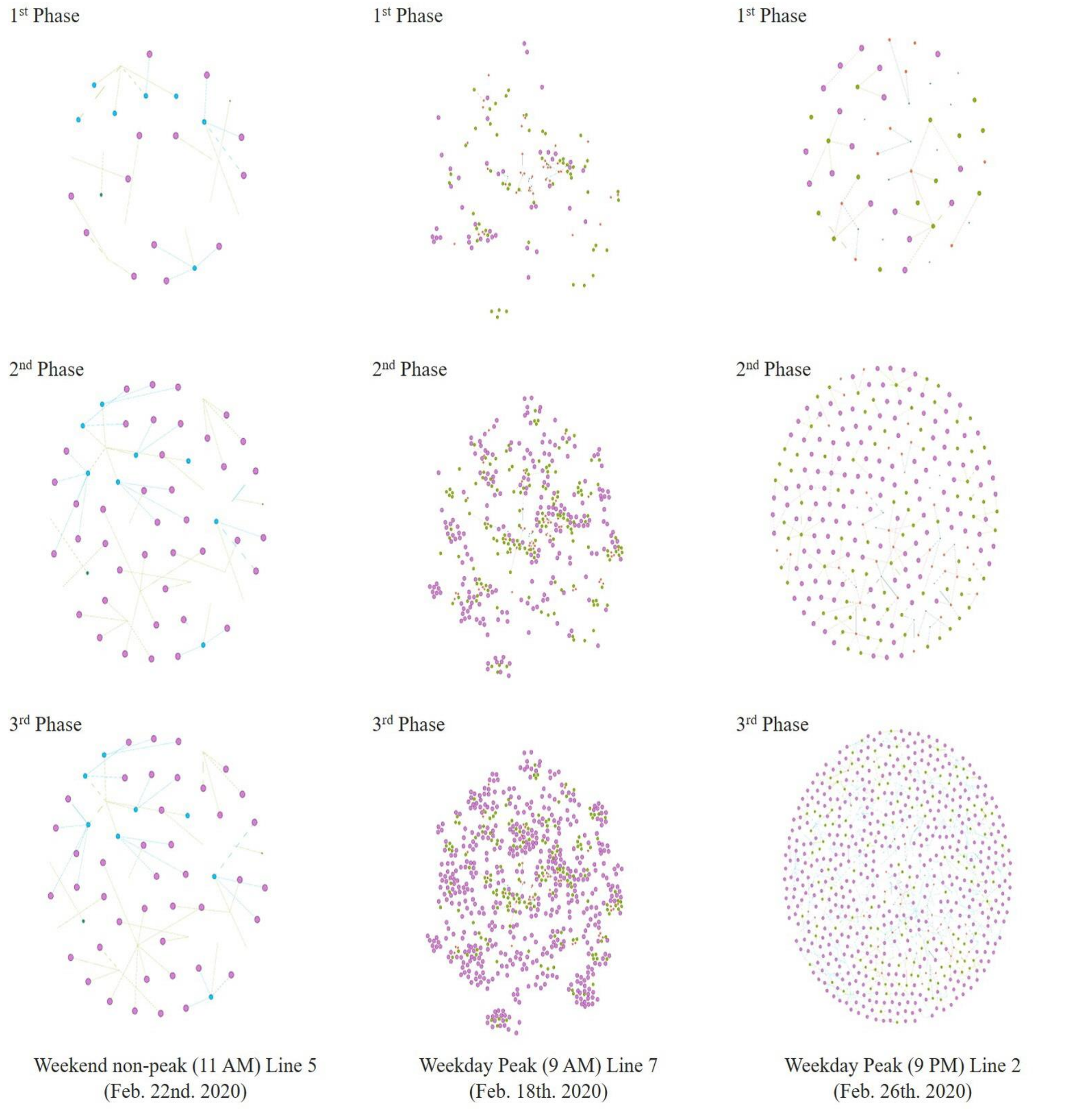

Fig. S13. Analysis of encounters by route, time, and actual infection route.

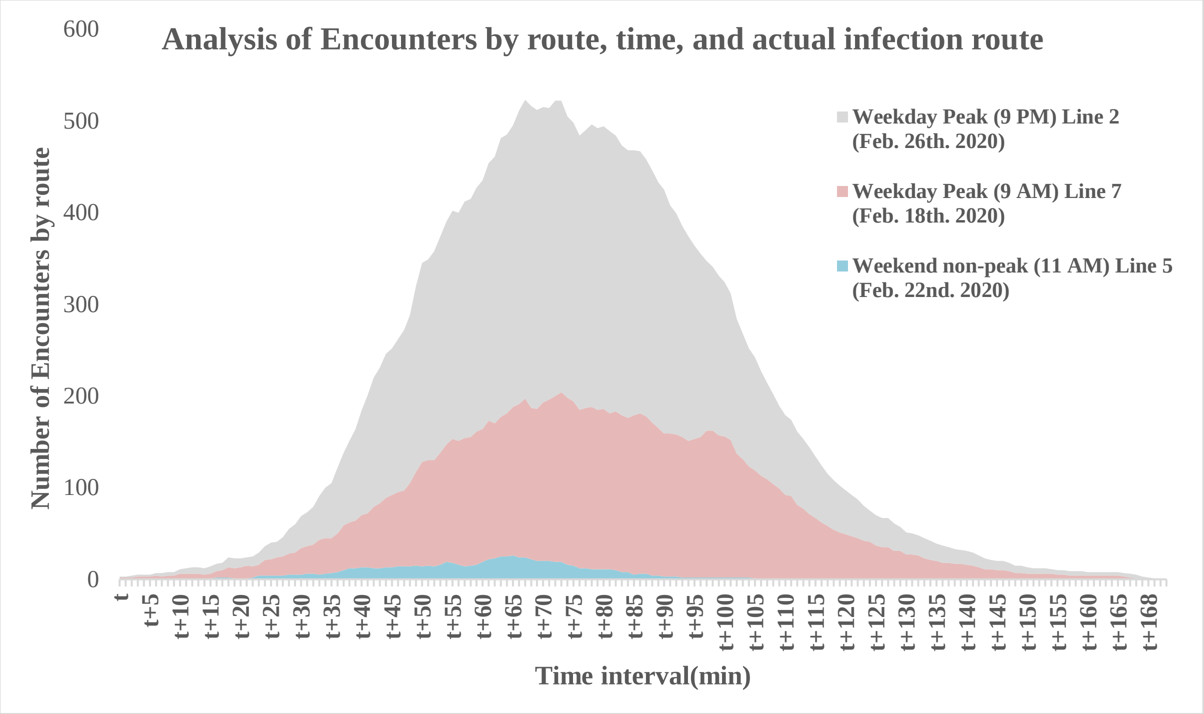

Fig. S14. Analysis of encounters by route, time, and actual infection route.

4. COVID-19 in South Korea

4.1 Model parameters and adjustment factors in this study

Currently, South Korea is responding to COVID-19 by implementing many measures, including the introduction of a work system with social distancing. Due to these measures, the actual rate of use of public transportation has sharply decreased. In this study, despite using smart card data, the congestion was recalculated by reducing the amount of public transport traffic with respect to time zone based on the actual reduced traffic ratio. Given that the degree of exposure in this study is closely related to the congestion in public transportation, the realistic infection risk in the current response situation is calculated based on the recalculated congestion, as shown in **Table S9.**

Table S9. Reduced traffic ratio between 2019 and 2020.

2019			2020			ratio
date	day	Number of Passengers	date	day	Number of Passengers	
2019-02-18	Mon	8,001,287	2020-02-17	Mon	7,178,330	-10.30%
2019-02-19	Tue	8,049,224	2020-02-18	Tue	7,210,230	-10.40%
2019-02-20	Wed	8,219,943	2020-02-19	Wed	7,286,559	-11.40%
2019-02-21	Thu	8,276,595	2020-02-20	Thu	7,154,541	-13.60%
2019-02-22	Fri	8,605,459	2020-02-21	Fri	7,015,803	-18.50%
2019-02-23	Sat	6,672,300	2020-02-22	Sat	4,036,339	-39.50%
2019-02-24	Sun	4,725,601	2020-02-23	Sun	2,634,575	-44.20%
Total		52,550,409	Total		42,516,377	-19.10%

Based on the analyzed encounter network, we estimated the diffusion model using the SEIR model in the current COVID-19 situation. We set variable values for exposure based on various research cases listed in **Table S10.** Furthermore, the variables in **Table S10** are variables that assume a special situation involving no masks. When the variable was applied, it was possible to confirm the process of exposure according to the degree of contact duration under.

Table S10. Parameters for the time-varying encounter network.

Category	Parameters	Value	Sources
Epidemic	β_I	0.0468	*(32)*
	β_E	$\beta_I \times 0.01$	*(7)*
	γ	0.0104	*(32)*
	μ_γ	4.17×10^{-4}	*(38)*
	μ_d	4.17×10^{-4}	*(38)*

4.2 "Uncontrolled" and "Controlled" situations in South Korea and Policy proposal

In this study, we used the encounter network analysis to verify the SEIR model for the case when actual infected individuals use public transportation without wearing a mask and no social distancing is maintained with others. We calculated the hazardous area using the model. However, in the current COVID-19 response situation, the countermeasure policies, such as wearing a mask and maintaining a social distance, are implemented. Due to these policies, the spread of infectious diseases has been significantly prevented. Specifically, it is considered that wearing a mask and maintaining social distance can significantly reduce infection in public transportation. We therefore performed additional analysis of a case study wherein it was assumed that individuals wore a mask and maintained social distancing in the current situation.

Figures A and B of **Fig. S15.** show the proportion of exposed and infected agents, respectively. This plot is obtained by applying the data to the public transportation network with respect to date based on the SEIR model. As shown in the graph, the exposed and infected agents are reduced when social distancing and wearing of masks are implemented.

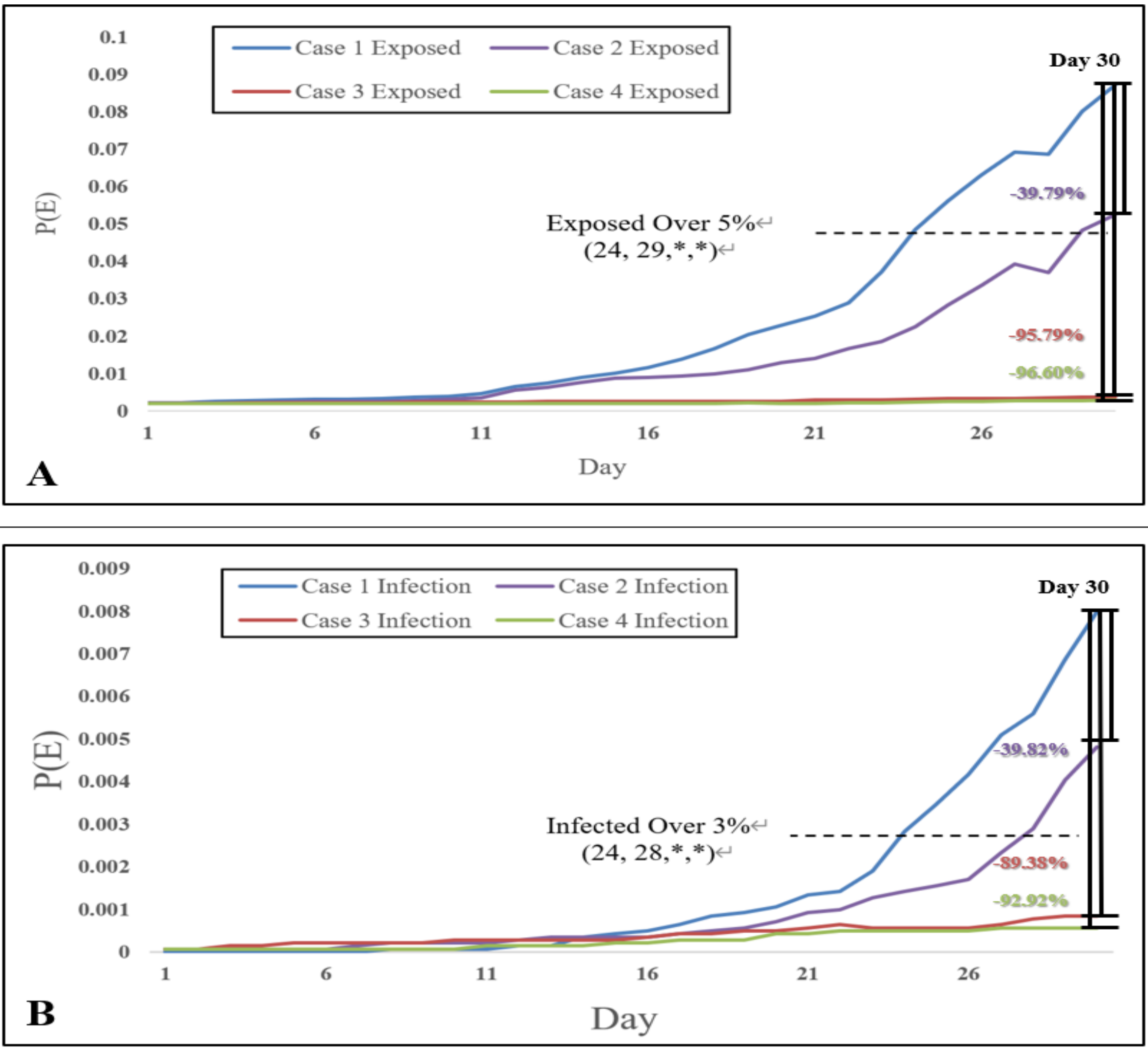

Fig. S15. Exposed and Infected trend based on the date of each case.

First, we use the public transportation smart card data for building a trip chain for passers-by and predicting individual route. Then, through the encounter network analysis, we identify the passengers who were exposed. By considering the case of wearing masks and congestion level, we apply the SEIR Model (especially exposed) to analyze the degree of exposure to the virus. Thus, it is possible to reduce exposure through two methods. Based on this, a social distancing policy can be proposed to provide detour routes from the user's side, adjust the operation schedule from the supplier's side, set the central quarantine section at the government level, and control the congestion of public transportation, as shown in **Fig S16.**

Table S11. Number of exposed agents in public transportation over time.

Days	P(Exposed)				Reduction in infection rate compared to Case 1		
	Case 1	Case 2	Case 3	Case 4	Case 2	Case 3	Case 4
1	0.21%	0.21%	0.21%	0.20%	0.0%	-3.3%	-6.7%
2	0.22%	0.21%	0.21%	0.20%	-3.2%	-6.5%	-9.7%
3	0.25%	0.22%	0.21%	0.20%	-11.4%	-17.1%	-20.0%
4	0.28%	0.23%	0.21%	0.20%	-17.9%	-23.1%	-28.2%
5	0.29%	0.24%	0.21%	0.20%	-17.1%	-26.8%	-31.7%
6	0.30%	0.25%	0.21%	0.20%	-18.6%	-30.2%	-34.9%
7	0.30%	0.25%	0.21%	0.20%	-16.3%	-30.2%	-34.9%
8	0.33%	0.26%	0.22%	0.20%	-21.3%	-34.0%	-40.4%
9	0.37%	0.28%	0.23%	0.20%	-23.1%	-36.5%	-46.2%
10	0.40%	0.32%	0.23%	0.20%	-19.6%	-41.1%	-50.0%
11	0.46%	0.35%	0.23%	0.20%	-24.6%	-49.2%	-56.9%
12	0.64%	0.55%	0.24%	0.20%	-14.3%	-62.6%	-69.2%
13	0.75%	0.63%	0.25%	0.20%	-16.0%	-66.0%	-73.6%
14	0.89%	0.77%	0.26%	0.20%	-13.5%	-70.6%	-77.8%
15	1.00%	0.88%	0.26%	0.19%	-12.0%	-73.9%	-81.0%
16	1.17%	0.91%	0.26%	0.19%	-22.4%	-77.6%	-83.6%
17	1.39%	0.93%	0.26%	0.21%	-33.0%	-81.2%	-85.3%
18	1.68%	0.99%	0.26%	0.21%	-40.9%	-84.4%	-87.8%
19	2.04%	1.10%	0.26%	0.22%	-46.0%	-87.2%	-89.3%
20	2.29%	1.29%	0.26%	0.21%	-43.8%	-88.6%	-91.0%
21	2.54%	1.41%	0.29%	0.21%	-44.6%	-88.6%	-91.9%
22	2.90%	1.67%	0.29%	0.22%	-42.4%	-90.0%	-92.4%
23	3.72%	1.86%	0.30%	0.23%	-50.0%	-92.0%	-93.9%
24	4.85%	2.25%	0.30%	0.24%	-53.6%	-93.7%	-95.0%
25	5.63%	2.84%	0.33%	0.25%	-49.5%	-94.2%	-95.6%
26	6.32%	3.37%	0.33%	0.26%	-46.8%	-94.9%	-95.9%
27	6.93%	3.93%	0.34%	0.28%	-43.4%	-95.1%	-96.0%
28	6.88%	3.70%	0.35%	0.28%	-46.2%	-95.0%	-96.0%
29	8.02%	4.84%	0.37%	0.28%	-39.7%	-95.4%	-96.5%
30	8.73%	5.26%	0.37%	0.30%	-39.8%	-95.8%	-96.6%

In case 1, social distancing and wearing of masks is not implemented. This causes a sharp increase in the number of infected cases on a daily basis since its initial generation. In case 2, the number of absolute agents decreased when the mask was not worn. However, the probability of the exposed agent was high, resulting in a certain amount of exposure agent and a 39.8% decrease after 30 days when compared to that in case 1. In case 3, social distancing was not performed. However, the probability of exposure was significantly reduced due to wearing a mask, resulting in a 95.8% decrease after 30 days when compared to that in case 1. In case 4, when both policies were implemented, the number of exposed agents did not change significantly, and the number of exposed agents differed significantly over time when compared to that in case 1, thereby showing a 96.6% decrease after 30 days, as shown is **Table S12.**

Table S12. Number of exposed agents under different cases during peak hour.

Congestion Level (%)	Interval (m)	Number of Exposed				Reduction in infection rate compared to Case1		
		Case1	Case2	Case3	Case4	Case 2	Case 3	Case 4
170-180	0.51	8,975	-	830	-	-	-90.8%	-
160-170	0.53	8,361	-	809	-	-	-90.3%	-
150-160	0.55	5,007	-	378	-	-	-92.5%	-
140-150	0.56	3,254	1,096	40	4	-66.3%	-98.8%	-99.9%
130-140	0.59	2,257	424	29	11	-81.2%	-98.7%	-99.5%
120-130	0.61	4,019	817	37	11	-79.7%	-99.1%	-99.7%
110-120	0.64	114	52	14	14	-54.4%	-87.7%	-87.7%
100-110	0.67	586	200	104	29	-65.8%	-82.3%	-95.0%
90-100	0.70	601	550	112	34	-8.4%	-81.3%	-94.3%
80-90	0.75	1,271	939	176	55	-26.1%	-86.2%	-95.7%
70-80	0.80	870	349	65	19	-59.9%	-92.6%	-97.8%
60-70	0.86	957	375	69	19	-60.8%	-92.8%	-98.0%
50-60	0.94	364	283	115	26	-22.3%	-68.4%	-93.0%
40-50	1.06	427	136	30	10	-68.1%	-93.0%	-97.8%
30-40	1.22	160	22	17	8	-86.6%	-89.4%	-95.3%
20-30	1.49	56	18	10	5	-67.6%	-82.0%	-91.0%
10-20	2.11	14	7	6	3	-50.0%	-57.1%	-78.6%
total		195,596	69,537	12,806	3,785	-64.4%	-93.5%	-98.1%

The table above shows the median of the exposed numbers according to the level of congestion in each case study during peak hours. If no policy was implemented, high levels of congestion and exposed numbers appeared in public transportation. In case 2, which implemented the social distancing policy, the reduction in infection rate was 64.4% higher when compared to that in case 1, which corresponds to no policy. This results in a decrease in the exposure of encounter degree and encounter duration as congestion level decreases. In case 3, which implemented the mandatory wearing of mask policy, the reduction in infection rate was 93.5% higher when compared to that in case 1. This led to a significant reduction in exposure due to the effect of wearing mask, which was derived from experiments. In this case, the congestion level was similar to that in case 1. Finally, when the two policies were implemented together, the reduction in infection rate was analyzed to be 98.1%, and thereby reflecting the effect of the reduction on congestion in public transportation and wearing of masks.

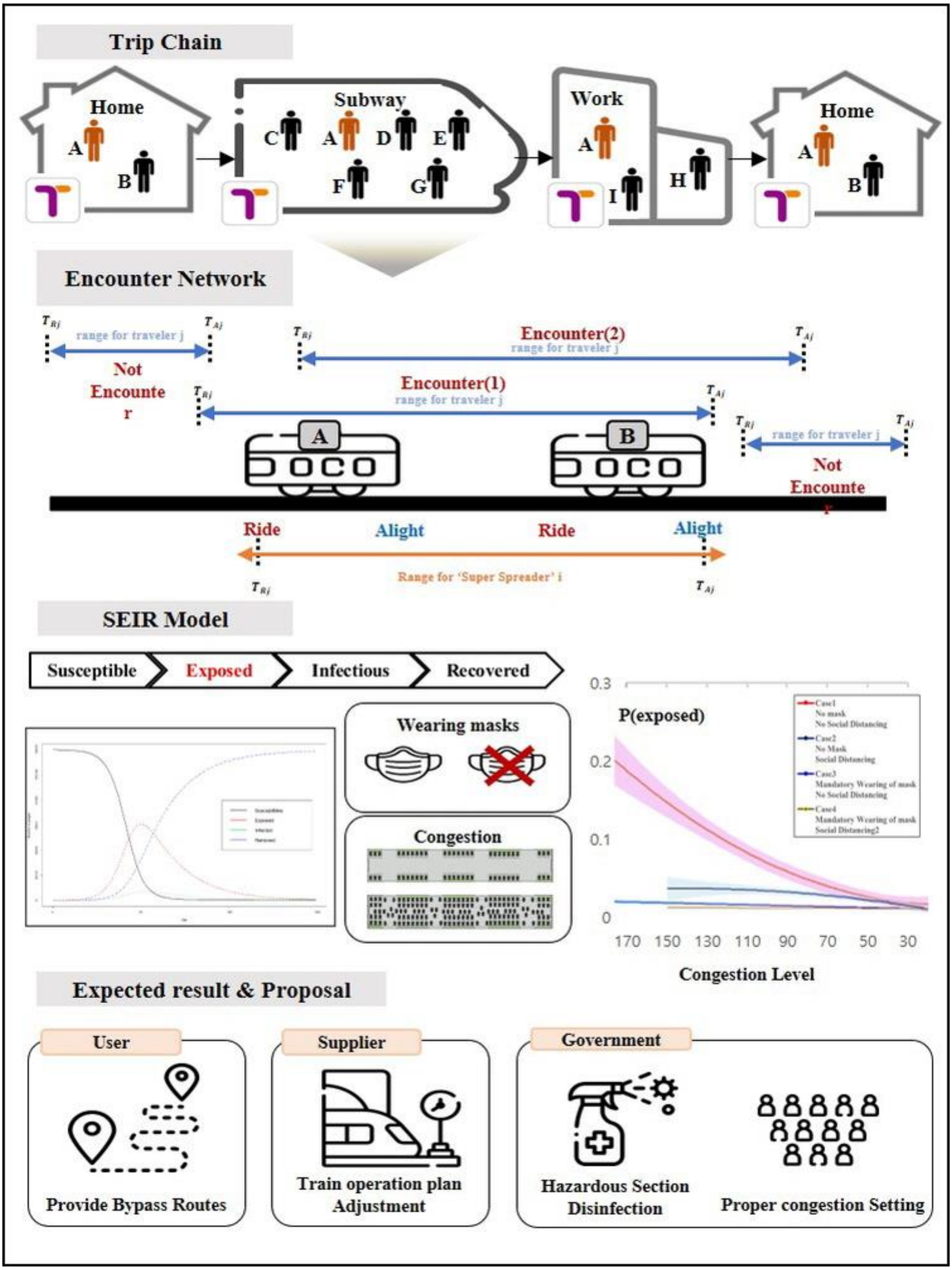

Fig. S16. Policy Proposal for the prediction of Spread Paths in Public Transportation.

Movie S1. Generating Exposed Agent when Infected passes on Line 2.

Movie S2. Generating Exposed Agent when Infected passes on Line 5.

Movie S3. Generating Exposed Agent when Infected passes on Line 7.

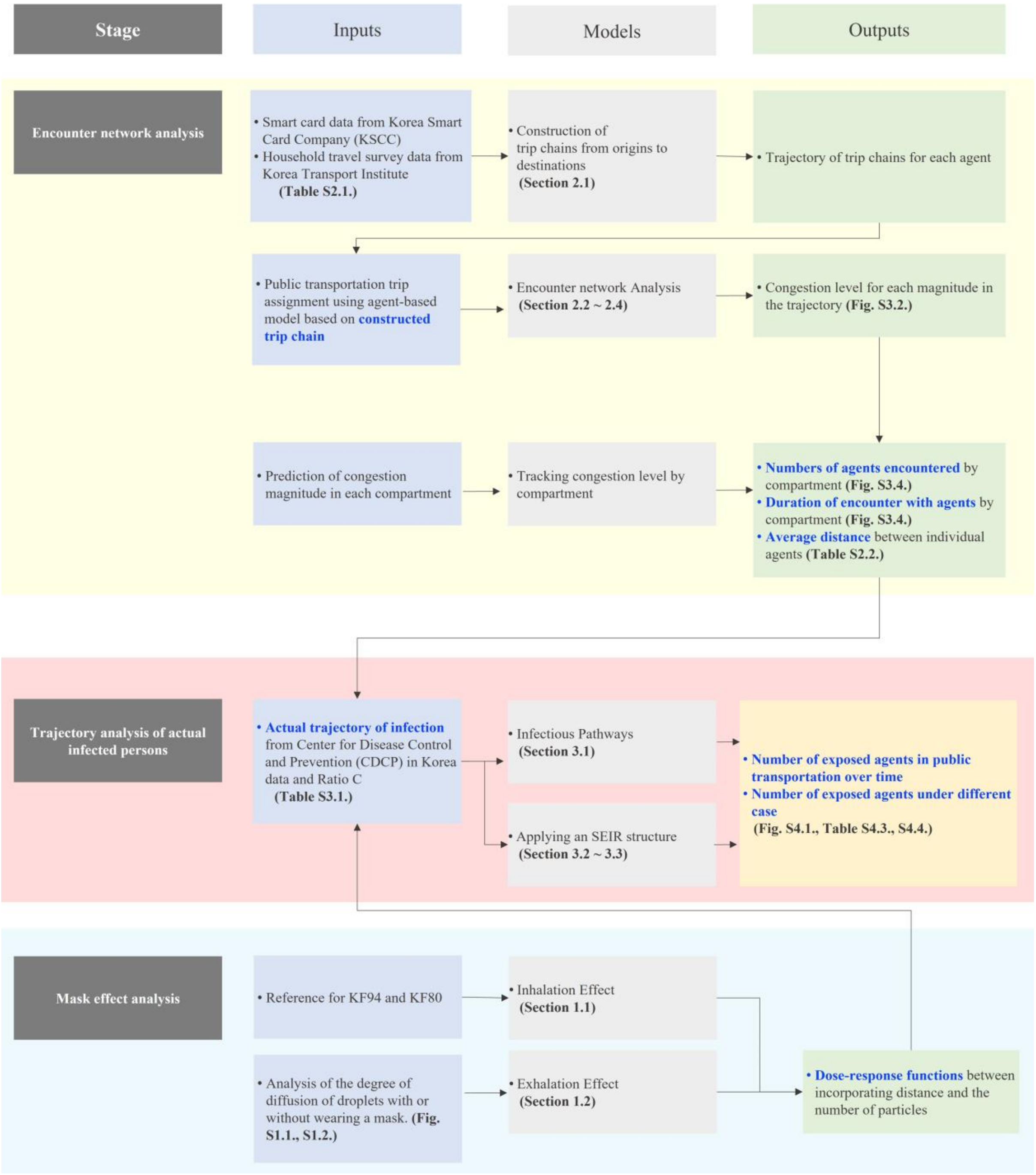

Fig. S17. A framework of modeling encounter analysis on trajectory infected data.
